# Multiple invasions of a generalist herbivore—Secondary contact between two divergent lineages of *Nezara viridula* Linnaeus in Australia

**DOI:** 10.1111/eva.12971

**Published:** 2020-06-22

**Authors:** Dean Robert Brookes, James P. Hereward, Lewis J. Wilson, Gimme Hugh Walter

**Affiliations:** ^1^ School of Biological Sciences The University of Queensland Brisbane QLD Australia; ^2^ Cotton Research Unit CSIRO Agriculture and Food Narrabri NSW Australia

**Keywords:** admixture, gene flow, microsatellite, mito‐nuclear discordance, phylogeography

## Abstract

The presence of distinct evolutionary lineages within herbivorous pest insect taxa requires close attention. Scientific understanding, biosecurity planning and practice, and pest management decision‐making each suffer when such situations remain poorly understood. The pest bug *Nezara viridula* Linnaeus has been recorded from numerous host plants and has two globally distributed mitochondrial (mtDNA) lineages. These mtDNA lineages co‐occur in few locations globally, and the consequences of their divergence and recent secondary contact have not been assessed. We present evidence that both mtDNA lineages of *N. viridula* are present in Australia and their haplotype groups have a mostly separate distribution from one another. The north‐western population has only Asian mtDNA haplotypes, and the population with an eastern distribution is characterized mostly by European mtDNA haplotypes. Haplotypes of both lineages were detected together at only one site in the north of eastern Australia, and microsatellite data indicate that this secondary contact has resulted in mating across the lineages. Admixture and the movement of mtDNA haplotypes outside of this limited area of overlap has not, however, been extensive. Some degree of mating incompatibility or differences in the climatic requirements and tolerances of the two lineages, and perhaps a combination of these influences, might limit introgression and the movement of individuals, but this needs to be tested. This work provides the foundation for further ecological investigation of the lineages of *N. viridula*, particularly the consequences of admixture on the ecology of this widespread pest. We propose that for now, the Asian and European lineages of *N. viridula* would best be investigated as subspecies, so that “pure” and admixed populations of this bug can each be considered directly with respect to management and research priorities.

## 1 INTRODUCTION

The association of insect pests with agriculture has facilitated the invasion of many of them outside their native range (Anderson, Tay, McGaughran, Gordon, & Walsh, 2016; Cristescu, 2015; Paini et al., 2016). Generalist host relationships, in particular, may facilitate invasion because of the greater number of host plants suitable for such organisms. Interpreting the invasion history and ecological features that underpin the geographical expansion of widely distributed insect species with varied ecology can be difficult. Understanding how the populations and ecology of an invasive species relate to populations found elsewhere in its distribution, however, is crucial for setting appropriate management and research priorities, as well as biosecurity measures.

Invasive populations may be comprised of once‐restricted geographic lineages that have come into secondary contact and may have experienced genetic admixture (Blackburn et al., 2011; Dlugosch, Anderson, Braasch, Cang, & Gillette, 2015; Garnas et al., 2016; Reil et al., 2018; Walther et al., 2009). Some invasive insects, once seen as one generalist species, have been found to represent a complex of cryptic species, with each one relatively more specialized in its host use (Dumas et al., 2015; Malka et al., 2018; Rafter, Hereward, & Walter, 2013). But genetic analysis has also revealed cryptic species complexes comprised of different generalist species (Gikonyo et al., 2017; Hereward, Hutchinson, McCulloch, Silva, & Walter, 2017; Vyskočilová, Seal, & Colvin, 2019). All of this implies that close attention should be paid when divergent evolutionary lineages have already been documented in a particular species. Quantifying the respective invasive distributions of such lineages, and their species status, is an important first step towards understanding the ecology of each and their further invasive potential and pest status.

Scientific understanding, biosecurity planning and management decisions may be compromised when multiple invasions are not clearly understood, especially when unresolved cryptic species complexes are involved (Walter, 2003). The practical implications may be substantial. For example, knowing that the *Scirtothrips aurantii* Faure (Thysanoptera) population (sensu lato) that established in Australia does not represent the economically damaging African citrus pest of that name, but a host specialist species within that complex (Rafter et al., 2013), means that quarantine precautions must continue to be strictly maintained. Insects that exhibit differences in host use across their invasive and native ranges, as seen in *S. aurantii* (Rafter et al., 2013), provide a clear signal that more than one species may be involved. However, when considerable overlap is evident in host use across evolutionary lineages (Hereward et al., 2017; Vyskočilová et al., 2019), detecting the presence of cryptic species becomes more difficult (Paterson, 1991; Walter, 2003). This is particularly relevant to generalist insect herbivores because the diversity of their host interactions tends to obscure any differences across species. Multiple invasions of such generalists are therefore more likely to remain undetected and uninvestigated, and so our understanding of these situations, and how to deal with them in practical terms, is likely to remain limited.

The biology and ecology of *N. viridula*, a globally distributed generalist bug, has been investigated for decades. The broad host plant range of *N. viridula* and the association of these insects with agriculture help explain its almost global distribution (Panizzi, 1997; Todd, 1989). However, three mitochondrial (mtDNA) lineages of *N. viridula* are known and their global distributions are mostly separate (Kavar, Pavlovčič, Sušnik, Meglič, & Virant‐Doberlet, 2006; Li et al., 2010). The European and Asian mtDNA lineages of *N. viridula* are distributed relatively widely but are reported to be sympatric only in Japan (Kavar et al., 2006). A highly divergent African lineage is restricted to Africa, but this lineage has been characterized genetically from only a single individual (Kavar et al., 2006; Li et al., 2010) and almost certainly represents a species distinct from the Asian and European lineages of *N. viridula* (Li et al., 2010). The consequences of secondary contact between the Asian and European lineages has not been investigated comprehensively.

Whether there are any ecological differences associated with each of lineages is not clear. Nevertheless, some geographically separate populations of *N. viridula* appear to exhibit distinct differences from one another that have caused several authors to raise the possibility that cryptic species may be present within *N. viridula* (Jeraj & Walter, 1998; Kavar et al., 2006; Ryan, Cokl, & Walter, 1996; Virant‐Doberlet, Čokl, & Stritih, 2000). For example, mating asymmetry is reported, as are differences in sexual sound communication across populations (Jeraj & Walter, 1998; Kon, Oe, Numata, & Hidaka, 1988; Ryan et al., 1996; Virant‐Doberlet et al., 2000). Differences in pheromone profiles have been detected in *N. viridula* from different locations (Aldrich, Oliver, Lusby, Kochansky, & Lockwood, 1987; Miklas, Renou, Malosse, & Malosse, 2000, but see Ryan, Moore, & Walter, 1995), and the photoperiod required to induce diapause may also vary (Todd, 1989). However, no morphological differences across populations have been detected, even in genital morphology (Ferrari, Schwertner, & Grazia, 2010; Qi, Walter, & O'Toole, 1999). Without investigating the biological characteristics mentioned above in relation to the mitochondrial lineages known to exist in *N. viridula*, this issue will remain unresolved. Such studies need to be conducted with a sound understanding of the genetic background of local *N. viridula* populations, particularly in countries where both mtDNA lineages are found as any admixture between the lineages will further complicate these investigations.

*Nezara viridula* feeds on plants from over 30 families, including many species of agricultural or horticultural significance, and all life stages of this insect feed preferentially on the fruit or seeds of their host plants (Todd, 1989). The bugs move through a sequence of host plants annually, as those hosts become more or less available and suitable for feeding and oviposition (Olson, Ruberson, Zeilinger, & Andow, 2011; Panizzi, 1997; Panizzi & Meneguim, 1989; Panizzi, Vivan, Corrêa‐Ferreira, & Foerster, 1996; Todd, 1989; Velasco & Walter, 1992), with regional populations and different generations using only a small subset of the entire host plant range of the species. At least one host plant species, *Ricinus communis,* does not appear to be equally suitable for *N. viridula* bugs of different provenance (Panizzi, 1997; Panizzi & Meneguim, 1989). *Nezara viridula* is invasive to Australia where it feeds mostly on crops and weeds (Velasco & Walter, 1992). In Australia, *N. viridula* has two principal generations (Velasco, Walter, & Harris, 1995), two fewer than is typical in the United States (Todd, 1989), and its abundance is frequently low during periods where few suitable host plants are available (Velasco & Walter, 1992; Velasco et al., 1995).

*Nezara viridula* has been present in Australia since at least 1911 (Clarke, 1992), and we present sampling and genetic analyses showing that both Asian and European mtDNA haplotypes are present on the continent. We investigated the distribution of the Asian and European *N. viridula* lineages in Australia by assessing the geographical distribution of their mtDNA haplotype groups. Microsatellites were then used to assess the amount of gene flow among individuals with mtDNA haplotypes of each lineage and among different geographic regions. We aimed to determine whether mating occurs between these lineages after secondary contact. We also conducted a global phylogeographic analysis of *N. viridula* using publicly available mtDNA sequences to compare Australian individuals with globally distributed samples of this species. Climatic factors affecting the distribution of each lineage in Australia are also explored. The implications for understanding and anticipating the outcome of multiple invasions and secondary contact in *N. viridula* are discussed. The results are then discussed within the context of the ecology and pest management of *N. viridula*, and their importance for generalist pest insects more broadly.

## 2 METHODS AND MATERIALS

### 2.1 Sampling and DNA extraction

A total of 649 individuals of *N. viridula* were sampled across Australia from 2014 to 2016, from 33 host plant species and across about 3,000 km (north to south) in eastern Australia, and also in two locations over 400 km apart in the north‐west of the continent. The colour morph (Kiritani, 1970) of each collected individual was noted. Most sampling locations were within agricultural regions where these insects are pests, and sampling sites within each sampling region were separated by at most 30 km. Crops and surrounding vegetation, including native and introduced plant species, were sampled at each site and while travelling between regions. Sampling involved sweep nets, beat sheets and extensive visual searching. The date, location, host plant and developmental stage (nymph or adult) were recorded for each individual. Collection localities, dates and host plant information are presented in Table S1. Collections were aggregated into regional samples for analysis, by region and date of collection, and are presented in Table 1.

**Table 1:** Populations of *Nezara viridula* (Pop.) from eight geographical regions of Australia

Pop.	Pop. Site Codes	*n*	Region	Lat.	Long.
KUN	GH01, KU01, KU02, KU03, KU04, KU05, KU06, KU07, KU08, KU09, KU10	118	North‐western	−15.672	128.725
DAR	DW01, DW02, DW03, DW04	29	North‐western	−12.510	131.107
LHR	LR01	2*	Far North QLD	−12.800	143.316
TOW	TV01	24	Northern QLD	−19.285	146.822
GRU	GU01	8*	Northern QLD	−19.571	147.137
BOW	BO01, BO02, BO03, BO04	48	Northern QLD	−20.052	148.161
EMRa	EM01, EM02	23	Central QLD	−23.549	148.202
EMRb	EM03, EM04	40	Central QLD	−23.549	148.202
BIL	BI01, BI02, BI04	56	Central QLD	−24.404	150.520
DAL	DA01, DA02, DA03, DA04	62	Southern QLD	−27.283	151.275
GAT	DA05, DA06, DA07	30	Southern QLD	−27.541	152.337
NARa	NA01, NA02	25	Central NSW	−30.255	149.553
NARb	NA04	24	Central NSW	−30.255	149.553
BBA	NA03	24	Central NSW	−30.542	150.010
BRZ	BR01, BR02, BR03	22	Central NSW	−31.186	150.433
GRIa	DP02, GR01, GR02	46	Southern NSW	−34.505	146.190
GRIb	GR04	25	Southern NSW	−34.441	146.037
HAY	HA01, HA02	23	Southern NSW	−34.472	144.753

Note

The population site codes represent the specific sites of collections, and they are grouped together into particular populations for analysis (see text for explanation and Table S1 for actual site details). The geographical region labels include state and territory abbreviations as follows: QLD, Queensland and NSW, New South Wales. Populations marked with an asterisk (*) were excluded from all analyses that did not group populations into regions because of small sample size. Latitude and longitude here represent the mid‐point between the sample sites that make up each designated population. The designated populations within geographical regions were at most 30 km from one another.

DNA extraction was performed on each *N. viridula* individual (mostly adults, see below), either by removing a leg and extracting with 20% Chelex solution (Bio‐Rad) or dissecting out flight muscle tissue for salt extraction (Supplemental Methods). The nymphs of *N. viridula* have limited mobility and are gregarious for some time after hatching from the egg mass (Todd, 1989), so adult insects were used to avoid introducing bias. When the sample size was small for a site and host combination, however, a maximum of one 4th or 5th instar nymph was included to bolster sample size.

### 2.2 Gene sequencing and analyses

The cytochrome C oxidase subunit I (COI) mitochondrial gene region was PCR‐amplified using primers LCO1490 and HCO21980 (Folmer, Black, Hoeh, Lutz, & Vrijenhoek, 1994) and sequenced to determine whether the Australian insects belonged to the Asian or European mtDNA haplotype groups of *N. viridula*. Mitochondrial sequences used in previous phylogenetic analyses (Kavar et al., 2006; Li et al., 2010) and from various published and unpublished sources were obtained as of 18 April 2018 (Table S2). Errors in the published COI sequences were assessed visually after aligning all sequences, and poor‐quality sequences were excluded where they could not be trimmed adequately. All sequences were aligned and trimmed for quality.

A phylogenetic analysis was performed on unique sequences from a 557‐bp fragment of the more usual COI barcoding region for 519 sequences, which included trimmed sequences from this study. A comparison was also made between sequences from this study and shorter sequences from the Kavar et al. (2006) data set to be certain no African lineage haplotypes were present in our sequences. These analyses were used to determine the relationship between Australian *N. viridula* and those found elsewhere, and not to supersede more comprehensive phylogenetic analyses that have been performed by other authors using more genetic data (Kavar et al., 2006; Li et al., 2010). To compare evolutionary models, Jmodeltest 2.1.4 (Darriba, Taboada, Doallo, & Posada, 2012; Guindon & Gascuel, 2003) was used together with corrected Akaike information criteria (AICc). The comparison showed the HKY (Hasegawa, Kishino, & Yano, 1985) evolutionary model with a Gamma (+G) distribution was the most appropriate under all likelihood criteria so it was used for the phylogenetic analysis. Maximum‐likelihood phylogenetic analysis was performed using the PhyML (Guindon et al., 2010) plugin as implemented in Geneious 9.0.5. Branch support was estimated using 5,000 bootstraps. Only sequences that were unique within each data set were used, and the COI sequence of *Eurydema gebleri* (Pentatomidae) (GenBank accession: KP207595.1) was included as the outgroup for the analysis.

Two nuclear DNA (nDNA) gene regions were also sequenced as previous studies used only mtDNA gene regions, and the evolutionary history of mtDNA is frequently not the same as nDNA (Toews & Brelsford, 2012). The tubulin alpha 1 (Tubα1) and the elongation factor 1 alpha (EF1α) gene region were sequenced for up to six Australian *N. viridula* from each host plant and site combination. Primers for both genes were developed de novo using Primer3 (Untergasser et al., 2012) based on the few sequences that amplified successfully using previously published primers (EF1α: Shirley and Prowler (Cho et al., 1995); Tubα1: TH_TubA forward and reverse (Buckman, Mound, & Whiting, 2013)). The presence of a microsatellite within the targeted EF1α gene region caused quality issues for many of the reverse primer sequences, so only the forward primer sequences could be used for many individuals (Table S1). To estimate the frequency of the nDNA haplotypes from diploid sequences, DNAsp 5.1 (Librado & Rozas, 2009) was used.

PCR protocols and primers for all gene sequencing are presented in Tables S3 and S4. PCR products were cleaned using Exonuclease I and Antarctic Phosphatase (New England Biolabs) and sequenced in both directions by Macrogen (Korea). Sequence data were trimmed, aligned and checked using CodonCode Aligner version 4.1.1 (CodonCode Corporation). Haplotype networks were created independently for the mitochondrial data, and for each of the two nuclear genes, using a TCS network (Clement, Posada, & Crandall, 2000) as implemented in PopArt (Leigh & Bryant, 2015) for all Australian *N. viridula*.

### 2.3 Microsatellite development and analyses

Microsatellite loci were used to assess the population genetic structure of Australian *N. viridula*. DNA from a single individual was shotgun‐sequenced at the Australian Genome Research Facility (AGRF) using the Illumina MiSeq platform. Paired end reads (250 bp) were merged using FLASh 1.2.7 (Magoč & Salzberg, 2011) for a total of 1,015,756 reads. The software QDD2 (Meglécz et al., 2010) was used to select potential microsatellite loci and design primers with Primer3 (Untergasser et al., 2012). Reads shorter than 80 bp, loci with fewer than five repeats, and dinucleotides were excluded outright. Primer pairs for 134 microsatellite loci were tested for amplification on individuals representing both the Asian and European lineages as determined by COI sequence data. The individuals used to develop these primers were sampled as geographically distantly from one another as possible in Australia, with the Asian lineage represented by insects collected from Kununurra in Western Australia, and the European lineage by insects over 2,500 km away from Griffith in New South Wales. The 63 loci that amplified across both lineages were tested again on population samples (23 individuals) of each lineage. The final 12 microsatellite loci were selected based on allelic richness and their low within‐lineage null allele estimates in initial populations.

Details of the microsatellite genotyping procedure can be found in Supplementary Methods. Peaks were confirmed and binned manually using the Geneious 9.0.5 microsatellite plugin 1.4.2. Individuals collected from nearby sites within sampled regions (defined in Table 1) were grouped into aggregate populations (i.e. regardless of host plant) for microsatellite analysis. These aggregate populations represent individuals collected at the same general location and time (Table 1). The 16 aggregate populations that had 19 or more genotyped individuals (see Table 1) were used to assess the suitability and quality of the microsatellite markers using summary statistics (Table 2). Null allele estimates and global F_ST_ were calculated using FreeNA (Chapuis & Estoup, 2007). Deviations from Hardy–Weinberg equilibrium (HWE) were measured using Hardy–Weinberg exact tests in Genepop 4.2 (Raymond & Rousset, 1995; Rousset, 2008), as were estimates of linkage disequilibrium. The number of private alleles was calculated using the *poppr* version 2.8.3 (Kamvar, Brooks, & Grünwald, 2015; Kamvar, Tabima, & Grünwald, 2014) for R (R Core Team, 2016).

**Table 2:** Statistics associated with the microsatellite data included in the population genetic analysis of Australian *Nezara viridula*

Locus	∑*N*a	$\hat{N}a$	*Ho*	*He*	HWE	Est. Null Alleles	g*F*_ST_
NEZA01	5	3.2	0.594	0.572	0	0.02	0.13
NEZA02	6	3.2	0.586	0.540	0	0.01	0.08
NEZA03	2	1.1	0.064	0.056	0	0.00	0.15
NEZA04	8	4.4	0.695	0.638	3	0.02	0.07
NEZA05	4	2.3	0.347	0.428	5 (3)	0.09	0.14
NEZA06	5	4.1	0.517	0.499	1	0.01	0.07
NEZA07	5	3.1	0.485	0.514	0	0.03	0.14
NEZA08	3	2.0	0.145	0.151	0	0.02	0.29
NEZA09	8	3.4	0.583	0.526	0	0.01	0.18
NEZA10	5	4.1	0.524	0.626	8 (3)	0.09	0.06
NEZA11	4	3.9	0.590	0.594	0	0.01	0.05
NEZA12	3	3.0	0.590	0.577	1	0.02	0.10

Note

Microsatellite summary statistics are the total number of alleles (∑*N*a), average number of alleles ( $\hat{N}a$), average observed heterozygosity (*Ho*), average expected heterozygosity *(He*) and deviations from Hardy–Weinberg Equilibrium (HWE) and within‐locus Bonferroni‐corrected HWE values shown in brackets. Null allele estimates and global *F*_ST_ are also shown. Only samples collected from the 16 populations with 19 or more individuals were used to generate these statistics (see Table 1), with the exception of the total number of alleles (∑*Na*) for which all could be included.

Microsatellite data were analysed using the individual‐based Bayesian clustering algorithm STRUCTURE 2.3.4 (Falush, Stephens, & Pritchard, 2003, 2007; Pritchard, Stephens, & Donnelly, 2000), which computes the probability of assignment for each individual to a chosen number of populations (K). We used Structure_threader (Pina‐Martins, Silva, Fino, & Paulo, 2017), STRUCTURE HARVESTER (Earl & vonHoldt, 2012) and CLUMPAK (Kopelman, Mayzel, Jakobsson, Rosenberg, & Mayrose, 2015). Population genetic structure could thus be assessed independent of any a priori population designations. STRUCTURE analyses were performed with the “admixture” and with the “correlated alleles” model. Values of K (assumed number of populations) from 1 to 10 were tested with 10 replicates for each K value. A burn‐in of 100,000 iterations, followed by 2,000,000 iterations, was used. The most appropriate K values were estimated using the Evanno method (Evanno, Regnaut, & Goudet, 2005). All K values that could be biologically meaningful are discussed, and they were used to direct further analyses (Porras‐Hurtado et al., 2013; Puechmaille, 2016). A principal components analysis (PCA) was performed as an alternative individual‐based assessment of the genetic structure of Australian *N. viridula*. PCAs were performed using the *adegenet* package (Jombart, Devillard, & Balloux, 2010) for R (R Core Team, 2016).

Pairwise population F_ST_ values were obtained in Genepop 4.2 (Raymond & Rousset, 1995; Rousset, 2008), with and without high null allele loci (~10%), to test whether these loci influenced the analyses unduly. Differences in allelic richness across the two gene pools that were identified by STRUCTURE were calculated manually, to compare their genetic diversity. Pairwise F_ST_ was used in Mantel tests to estimate isolation by distance (IBD) using the mantel.randtest function in the ade4 package 1.7 (Dray & Dufour, 2007) in R, with 1,000 permutations and using *F*_ST_/1‐*F*_ST_ as the measure of genetic distance and the log of geographic distance as per Rousset (1997). The IBD analyses were performed with (a) all populations, and (b) with only populations from eastern regions (excluding those from north‐western Australia). Eastern Australian populations were analysed separately because the sites are spatially more continuous, and the north‐western region is a geographic outlier. When two populations had been collected from the same sample location at different times (Table 1), the population sampled at the earliest time was used and so 89 individuals from populations EMRb, NARb and GRIb were excluded from IBD analyses.

### 2.4 Modelling climatic suitability

Climatic limits to the distribution of the lineages of *N. viridula* were modelled using MaxEnt version 3.4.1 (Phillips, Dudík, & Schapire, 2020) as implemented in BCCVL (Hallgren et al., 2016). MaxEnt was chosen because it predicts the largest possible range size from the available data (Merow, Smith, & Silander, 2013). Presence data were compiled from the sites sampled in this study and from nearby insect collection records obtained from Atlas of Living Australia, 2020 (ALA) with duplicate spatial records removed. Presence data were divided into two groups, (a) records from 55 localities with European mtDNA haplotypes and (b) records from 10 localities with Asian mtDNA haplotypes. Regions with both mtDNA haplotypes were included in both groups. Climatic data were obtained from the CliMond Bioclimate Map Time‐Series 1975 (Kriticos et al., 2012). The distribution of the European mtDNA lineage should be limited by its intolerance to high temperatures and high humidity based on experimental evidence from eastern Australia *N. viridula* (Chanthy, Martin, Gunning, & Andrew, 2015; Velasco & Walter, 1993). Mean moisture index for the warmest quarter and mean temperature for the warmest quarter were thus used as predictor variables. The model was geographically constrained to Australia so that background samples covered areas where both presence and absence were expected (Merow et al., 2013; Saupe et al., 2012). Receiver operating characteristic (ROC) curve was used to assess the suitability of the model.

## 3 RESULTS

### 3.1 Distribution of *Nezara viridula* lineages and secondary contact in Australia

**Figure 1:**
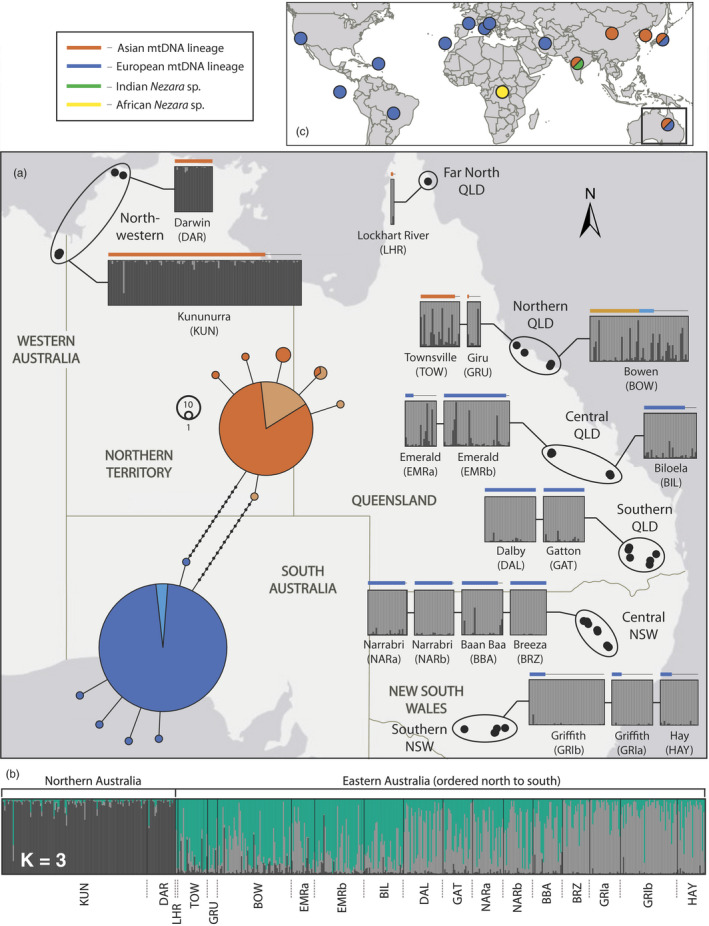
(a) Map of northern and eastern Australia with *Nezara viridula* sampling sites represented by black dots. The circles that surround sets of black dots amalgamate these nearby sampling sites into labelled regions (Table 1). Superimposed on the map is a haplotype network showing the relationship between the COI haplotypes of 480 Australian *N. viridula* individuals (all sequences), and also a K = 2 STRUCTURE analysis of the microsatellite data from 571 individuals. Horizontal bars above these locality‐specific inset boxes indicate the mitochondrial haplotype of the corresponding individual in the STRUCTURE analysis boxes below the bar. Those individuals that were genotyped, but which did not have their COI gene sequenced, are represented above the boxes by a thin black line. Bowen is the only location in which bugs with both mitochondrial haplotypes were found together, with a lighter shade of either orange or blue to distinguish bugs from that location from others. (b) A K = 3 STRUCTURE analysis is also shown below the map. (c) A global inset map is used to show the placement of all publicly available *N. viridula* sequences (Figure S1)

A total of 13 COI haplotypes were found from 480 Australian *N. viridula* individuals and 608 bp of the COI gene region. Seven haplotypes were associated with the Asian mtDNA haplotype group and six with the European mtDNA haplotype group. Individuals within each haplotype group mostly shared a single haplotype, 84% and 98% for the Asian and European haplotype groups, respectively (Figure 1). All Australian *N. viridula* mtDNA haplotypes were clearly associated with the Asian or European mtDNA lineages described previously (Kavar et al., 2006) (Figure S1). These mtDNA haplotype groups have a mostly separate distribution from one another in Australia (Figure 1; see coloured bars above STRUCTURE plots). The Asian mtDNA haplotype group is found in the north of Australia, including the northern parts of eastern Australia. The European mtDNA haplotype group is present in the central and southern parts of eastern Australia. The two mtDNA haplotype groups were found to be sympatric in only a single Australian location, Bowen, in northern Queensland (Figure 1). At this site, COI haplotypes belonging to the Asian mtDNA haplotype group were most common (81%). Two *N. viridula* clades present in publicly available sequences were not found in Australian individuals, the African clade described by Kavar et al. (2006) and a previously undocumented clade comprised entirely of sequences originating from India (Figure 1, Figure S1).

**Figure 2:**
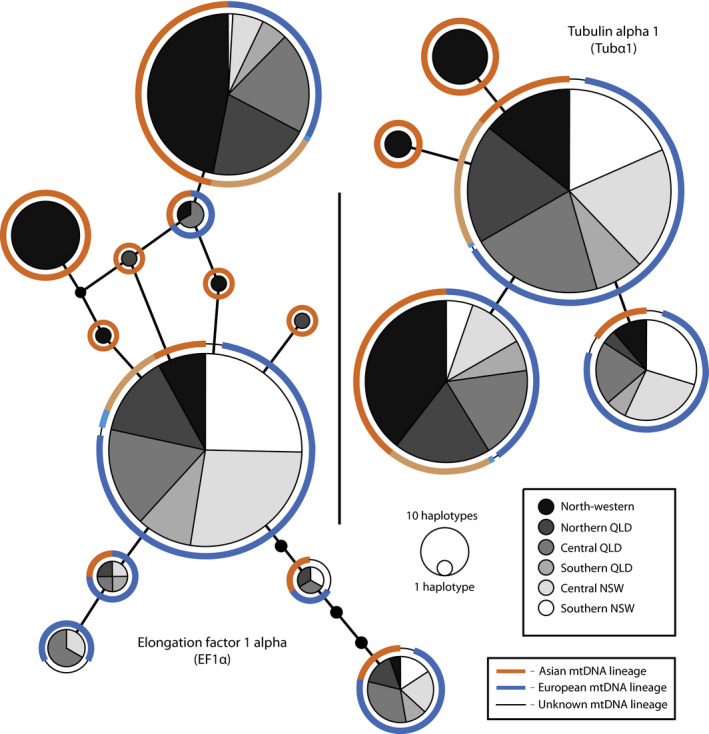
Haplotype networks for the EF1α (440bp) and Tubα1 (423bp) nuclear genes sequenced for individuals of *Nezara viridula* collected in Australia. Haplotype shade (ordered stepwise geographically from black (north) to white (south)) indicates the number of haplotypes obtained from each region (Table 1). For each region that contributes to the number of bugs with a particular EF1α or Tubα1 haplotype, the colour (blue or orange) of the circle around that haplotype corresponds to the individuals associated with each mitochondrial lineage (see key). Each individual is represented by two haplotypes as estimated by DnaSP 5.1 (Librado & Rozas, 2009). EF1α and Tubα1 haplotypes associated with both mtDNA lineages in the same region are indicated with a lighter shade of either orange or blue to distinguish th`em from those with a single regional mtDNA lineage

The most common nuclear gene haplotypes in Australian *N. viridula*, for both Ef1α and Tubα1, were present in insects from both of the mitochondrial lineages and so neither nuclear gene showed evidence that distinct nuclear gene pools exist within Australian *N. viridula* (Figure 2). Tubα1 had a total of five haplotypes across 423 bp and 177 individuals. The three most common Tubα1 haplotypes (95%) were present in both mitochondrial lineages, and the remaining two were found only in individuals with mtDNA of the Asian lineage. Ef1α was more diverse with 12 haplotypes across 440 bp and 167 individuals, but the two Ef1α haplotypes with the highest frequency (83%) were associated with both of the mitochondrial lineages. Of the remaining Ef1α haplotypes, one was associated only with the European mtDNA lineage, five only with the Asian lineage, and the other four were associated with both mtDNA lineages. Of the haplotypes unique to a single mtDNA lineage, only one of six was associated exclusively with the European mtDNA.

### 3.2 Gene flow across mtDNA lineages in Australian *Nezara viridula*

Microsatellite genotype data were obtained for 571 Australian *N. viridula* individuals at the 12 microsatellite loci used in this study. During development, several microsatellite loci were rejected because of the high estimate of null alleles (>20%) in populations representative of one or both lineages. Some of the 12 loci used to genotype Australian *N. viridula* showed significant departures from HWE, even after sequential Bonferroni correction within loci. In each case, this departure from HWE was not observed in all populations. No loci showed evidence for linkage disequilibrium. Two of the 12 loci, NEZA05 and NEZA10, had higher null allele estimates than all other loci, and although neither locus had average null allele estimates >10% across populations, these same two loci showed the most frequent departures from HWE (Table 2). All microsatellite analyses were thus performed a) with all 12 loci and b) with loci NEZA05 and NEZA10 removed. For pairwise *F*_ST_, only the southern populations of *N. viridula* (from Griffith and Hay, Figure 1) showed a change from significant to nonsignificant differences after these loci had been excluded (Tables 3 and Table S5).

**Table 3:** Pairwise F_ST_ values for 16 Australian populations of *Nezara viridula* based on 12 microsatellite loci

	KUN	DAR	TOW	BOW	EMRa	EMRb	BIL	DAL	GAT	NARa	NARb	BBA	BRZ	GRIa	GRIb	HAY
KUN	–	***	***	***	***	***	***	***	***	***	***	***	***	***	***	***
DAR	0.021	–	***	***	***	***	***	***	***	***	***	***	***	***	***	***
TOW	0.108	0.138	–	*	ns	**	*	***	***	***	***	***	***	***	***	***
BOW	0.140	0.169	0.011	–	ns	ns	ns	***	***	**	***	***	***	***	***	***
EMRa	0.144	0.179	0.012	−0.005	–	ns	ns	ns	*	ns	*	ns	***	***	***	***
EMRb	0.143	0.187	0.019	0.004	0.006	–	ns	***	***	**	***	***	***	***	***	***
BIL	0.150	0.184	0.012	−0.003	−0.014	0.002	–	*	*	*	*	***	***	***	***	***
DAL	0.204	0.234	0.049	0.021	0.009	0.032	0.011	–	ns	ns	ns	*	**	***	***	*
GAT	0.184	0.226	0.056	0.024	0.016	0.029	0.012	0.000	–	ns	*	***	***	***	***	**
NARa	0.190	0.230	0.039	0.015	0.012	0.014	0.008	−0.005	−0.005	–	ns	ns	*	**	**	**
NARb	0.195	0.238	0.034	0.027	0.012	0.027	0.007	0.008	0.013	0.003	–	*	*	***	***	**
BBA	0.188	0.232	0.053	0.022	0.006	0.031	0.017	0.007	0.015	0.004	0.011	–	**	***	***	**
BRZ	0.262	0.318	0.102	0.073	0.049	0.075	0.054	0.016	0.030	0.014	0.015	0.023	–	ns	ns	**
GRIa	0.265	0.332	0.114	0.071	0.050	0.073	0.062	0.021	0.036	0.024	0.035	0.024	−0.004	–	ns	ns
GRIb	0.267	0.323	0.116	0.080	0.070	0.072	0.071	0.033	0.039	0.026	0.047	0.044	0.021	0.015	–	*
HAY	0.225	0.281	0.062	0.045	0.037	0.039	0.035	0.011	0.029	0.016	0.016	0.017	0.024	0.009	0.022	–

Note

Two populations were excluded because of low sample size; *n* < 19 (Table 1). The results of pairwise exact G tests for genotypic differentiation are shown above the diagonal, with levels of significance: *0.01–0.05, **0.01–0.001, ***≥0.001. If more than one nearby location is included in a single population, the average pairwise geographical distance was used.

**Figure 3:**
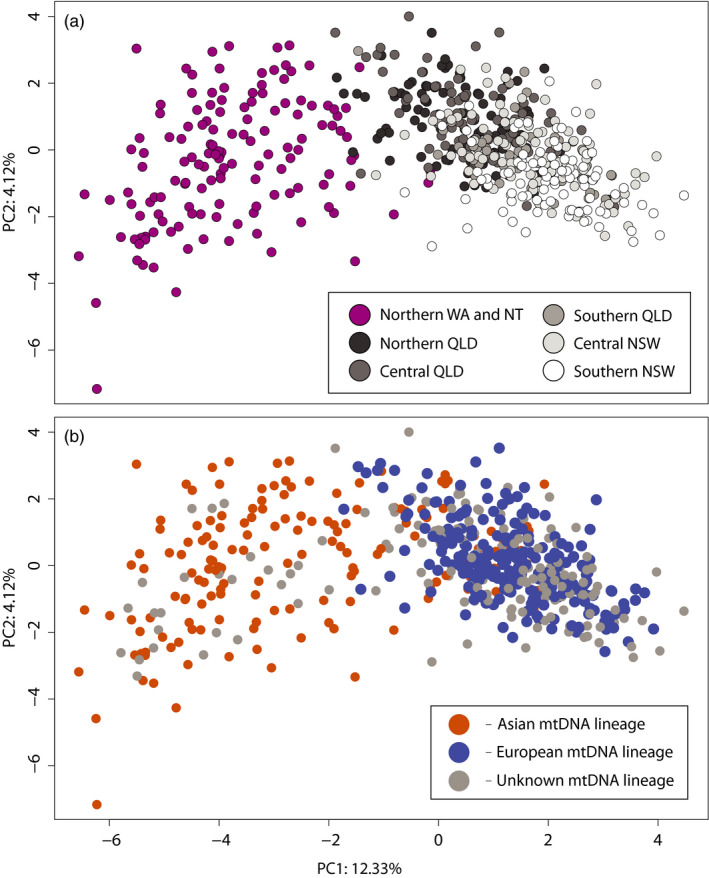
A principal components analysis (PCA) using microsatellite genotype data from the 10 loci without high null allele estimates, and from all *Nezara viridula* populations from Australia. Individuals are coloured according to: (a) their region of origin and (b) in relation to their mitochondrial haplotype group. Regional abbreviations are: NSW,  New South Wales; NT, Northern Territory; QLD, Queensland; WA, Western Australia

For the STRUCTURE analysis of the microsatellite data, the most likely inferred value of K was 2 (see Figure S2 for the likelihood of K and ΔK). The eastern populations of *N. viridula* cluster together in this K = 2 analysis, and are separate from those populations further west in northern Australia (from Kununurra and Darwin). The analysis, most importantly, shows the discordance between the microsatellite and mtDNA data in eastern Australia, with gene flow between individuals with mtDNA belonging to both lineages (Figures 1 and 3). The private allele analysis shows the same pattern (Table S6). The north‐western region had 15 private alleles from 185 individuals when compared to one private allele in a single individual for northern QLD and no private alleles for southern NSW. Other regions were excluded from the analysis so that an equivalent spatial could be made. Although eastern *N. viridula* individuals represent a single interbreeding population (STRUCTURE analysis in Figure 1), in the K = 2 STRCUTURE analysis, individuals from northern Queensland and central Queensland were more frequently assigned to the same population as north‐western individuals than were individuals from southern Queensland and New South Wales (Figure 1). This was the same for the K = 3 analysis, but fewer eastern individuals were assigned to the same population as north‐western individuals (Figure 1). All 12 loci were included in this analysis as the presence of null alleles should have only a minor impact on assignment tests (Carlsson, 2008).

**Figure 4:**
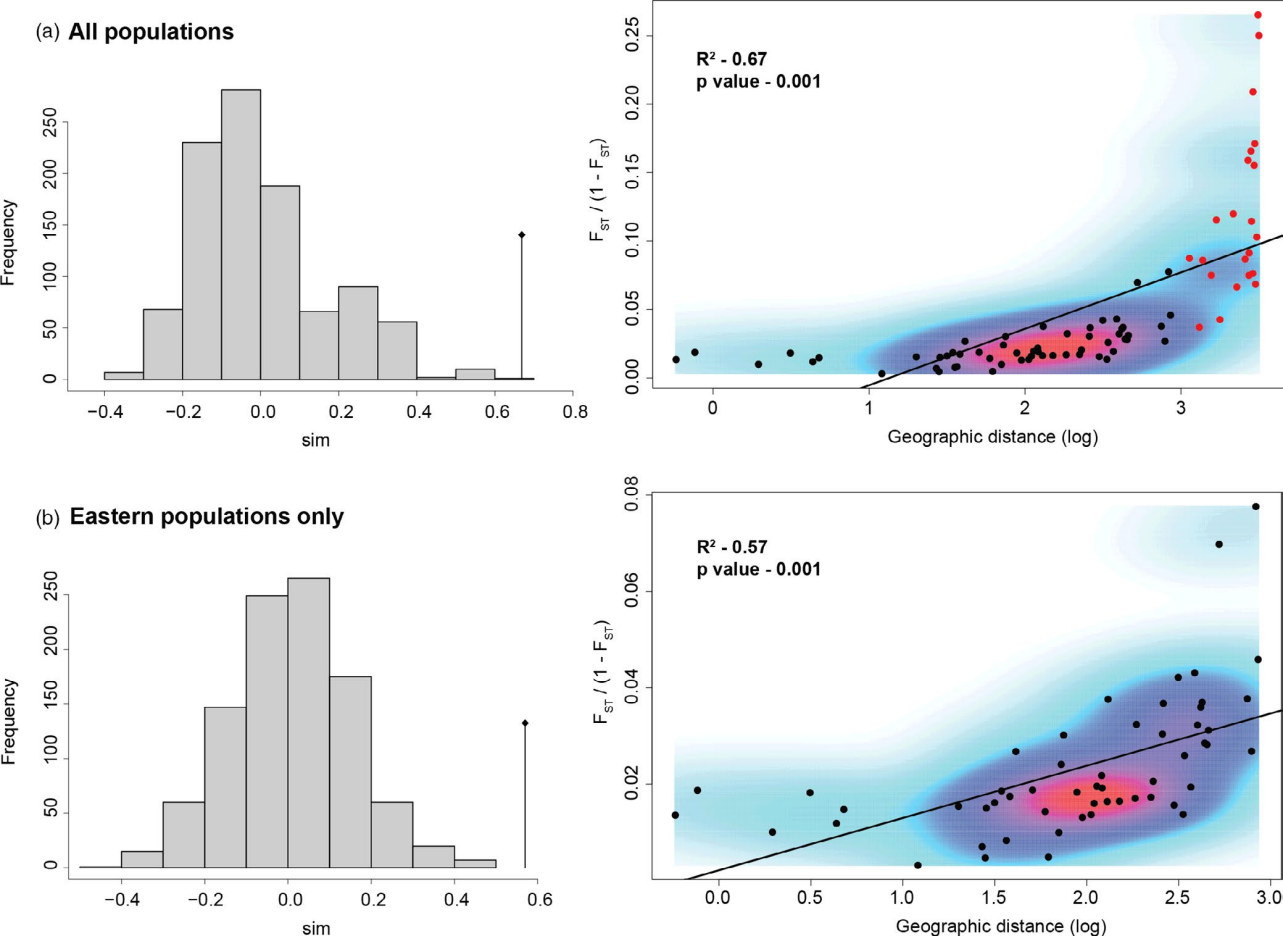
Two isolation‐by‐distance (IBD) relationships based on mantel tests are shown for *Nezara viridula* in Australia. The histograms summarize the result of mantel test simulations. Sites with fewer than 20 individuals, and those sampled a second time, were not included. All Australian *N. viridula* populations that fit this criteria are included in the top histogram and figure (a) but only the eastern Australian populations are included in the histogram and figure below (i.e., excluding the Darwin and Kununurra samples, (b)). The red points in figure (a) are pairwise comparisons between the Darwin and Kununurra samples and all eastern samples

Including loci with high null allele estimates introduced artefacts in the PCA and so the loci NEZA05 and NEZA10 were excluded from the final PCA. The northern populations from eastern Australia (e.g. TOW, Figure 1) are genetically much more similar to the north‐western (Asian mtDNA) populations than are the southern populations in eastern Australia (e.g. GRIa and GRIb, Figure 1) to the north‐western populations. This genetic relationship is shown by the individual population assignment in STRUCTURE (Figure 1), population clustering in PCA (Figure 3) and pairwise population *F*_ST_ (e.g., Townsville versus Kununurra is 0.108 while Griffith (GRIa) versus Kununurra is 0.265, Table 3). Isolation‐by‐distance analyses indicated some relationship between genetic distance and the geographic separation of populations, with R^2^ values of 0.67 for all populations and 0.57 when the north western populations were excluded from the analysis (Figure 4). However, IBD analyses also show that the relationship between genetic and geographic distance is not linear, whether the two north‐western populations are included (Figure 4a) or excluded (Figure 4b).

Finally, two minor points are worth mentioning. Specific host plant species could not be sampled frequently enough across different regions within the broad distribution of *N. viridula* to allow for a statistical analysis of genetic differentiation across host plants (in part because of drought). Instead, individual‐based clustering analyses (STRUCTURE and PCA) were used to infer that no host‐associated genetic structure was evident. The G, O, F and R colour morphs (Kiritani, 1970) of *N. viridula* were found in the north‐western Australian populations, while only the G colour morph (Kiritani, 1970) was found in eastern Australia.

### 3.3 Climatic suitability

**Figure 5:**
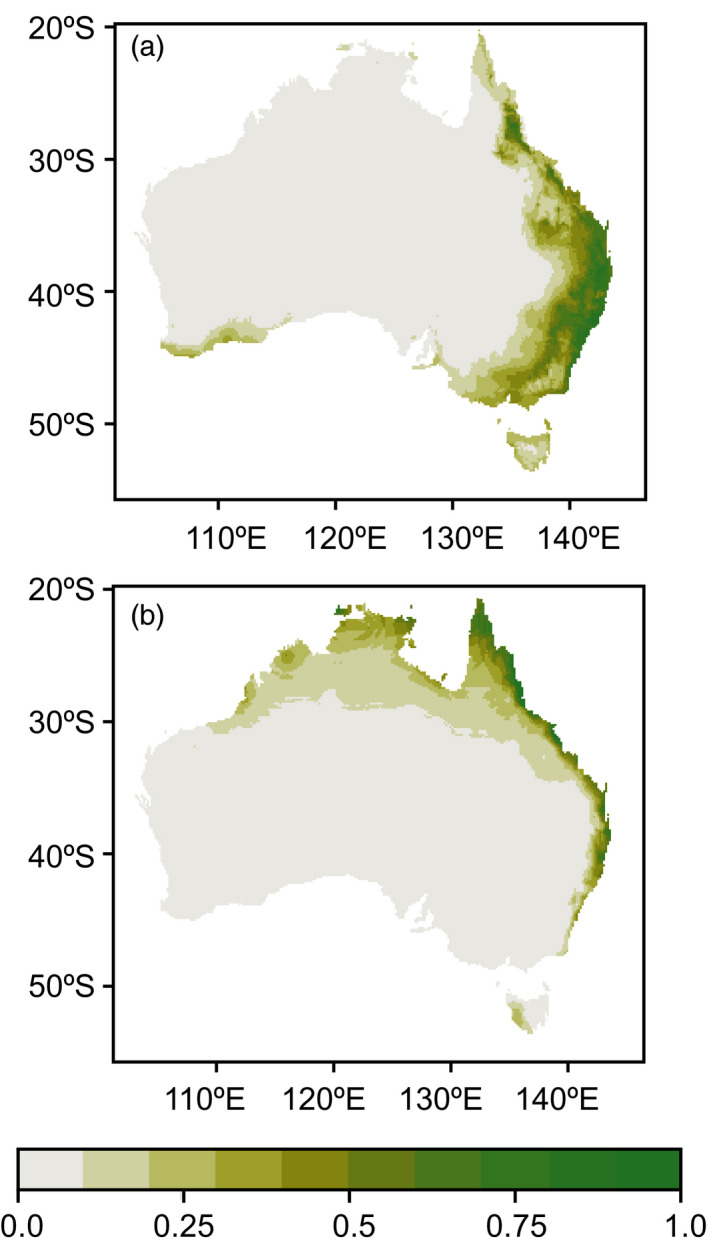
Climatic suitability modelled using (a) individuals with European mtDNA haplotypes and (b) individuals with Asian mtDNA lineage haplotypes. The one site with both mtDNA haplotypes (Bowen) was included in both groups. Occurrence records from Atlas of Living Australia (ALA) were also included from regions similar to those sampled in this study. Climate suitability was modelled using MaxEnt with mean moisture index for the warmest quarter and mean temperature for the warmest quarter used as predictor variables

The north‐western region, where only bugs with Asian mtDNA are found, was predicted to be unsuitable for individuals with European lineage mtDNA in the warmest annual quarter (Figure 5a). Much of the Australian east coast was predicted to be suitable during the warmest quarter for individuals with Asian mtDNA (Figure 5b). ROC values for both MaxEnt analyses were high (0.94 and 0.97, Figures S3 and S4) indicating good support for the model. Mean temperature and mean humidity for the warmest quarter were both good predictors of climatic suitability for *N. viridula* with European mtDNA (Figure S5). Mean temperature for the warmest quarter was the best predictor for climatic suitability of *N. viridula* with the Asian mtDNA haplotype and mean humidity contributed little (Figure S6).

## 4 DISCUSSION

### 4.1 Genetic structure and origin of Australian *N. viridula* populations

*Nezara viridula* populations in Australia, as well as globally, mostly belong to the European mtDNA haplotype group (Figure 1 and Figure S1). The mitochondrial data presented here reveal that the Asian and European mtDNA lineages of *N. viridula* are known to co‐occur only in Australia and Japan, though they may also be found together elsewhere. Secondary contact between the Asian and European lineages of *N. viridula* in Australia has resulted in cross‐mating and the production of offspring, as evidenced most clearly by the discordance between the mtDNA and nuclear (microsatellite) data from insects collected at Bowen (Figure 1). Mating between the Asian and European lineages in Australia has, however, not resulted in complete admixture or the widespread distribution of both mtDNA haplotype groups, as has been found other invasive insects in Australia (Toon et al., 2016). Importantly, the admixture across the Asian and European lineages means that the mtDNA haplotype group of an individual may not be representative of its nuclear genetic history.

Two genetically differentiated populations of *N. viridula* are found in Australia based on microsatellite data (Figure 1). The largely independent geographical distributions of the populations, each associated primarily with the Asian or European mtDNA haplotype groups, suggests that each lineage arrived separately to one another and that they remain largely separate today despite some admixture. One population is in the north‐western region (Kununurra and Darwin, Figure 1) and corresponds mostly to the Asian lineage of *N. viridula*. The other population is in eastern Australian regions and is mostly associated with European lineage bugs (all other regions, Figure 1). The number and abundance of private alleles indicates that north‐western region individuals differ in their allelic diversity when compared to populations in eastern regions (Tables S6 and S7). Individuals from southern NSW and northern QLD, by contrast, had almost identical allelic diversity (Tables S6 and S7). Three of the four colour morphs found in Australian *N. viridula* are associated only with bugs from the north‐western regions, providing further evidence for limited movement of insects between these regions. Some amount of mating probably occurs between these two populations, but further tests are required to determine whether mating between the lineages occurs at a lower frequency that within‐lineage mating.

Where mtDNA haplotype groups of both lineages are found together in northern Queensland, a bias towards the European lineage in the microsatellite data is clear, even when mtDNA haplotypes of the Asian lineage are more common (Figures 1 and 3), and even when geographic distance has been accounted for (Figure 4). Asian lineage mtDNA haplotypes are not found south of the northern QLD region, despite mating occurring between bugs of both lineages in sympatry, and despite *N. viridula* being a capable flier (Gu & Walter, 1989; Kester & Smith, 1984; Kiritani & Sasaba, 1969). The lack of Asian mtDNA haplotypes further south must be explained, as there are no obvious impediments to movement between the regions in which bugs of both mtDNA haplotype groups co‐occur and regions further south which have only European mtDNA haplotypes (northern QLD and central QLD, respectively; Figure 1). The genetic resolution of the nuclear gene fragments was low overall and because of this generally uninformative. The most common haplotypes are likely shared because of incomplete lineage sorting given that few substitutions separate them, although admixture between the lineages might also have play a role.

Biogeographical barriers to gene flow such as regional host availability, and possible ecological differences across the Asian and European lineages of *N. viridula*, might account for the presence of two genetically different populations and the restricted distribution of each mtDNA haplotype group. Limited host plant availability intervening the north‐west region and the eastern regions may reduce the number of bugs moving between them. In Australia, *N. viridula* feeds mostly on crops and introduced weed species (Velasco & Walter, 1992), and these are likely to be scarce in dry regions, including the gaps between sampled regions, and so not sustain *N. viridula* populations year‐round. Host availability may be higher in wet years and facilitate the movement of individuals between populations that are regularly isolated from one another by dry conditions. Host plant distribution does not explain completely why the distribution of each mtDNA haplotype group is restricted in eastern Australia. Some degree mating incompatibility (Jeraj & Walter, 1998; Ryan et al., 1996), population ephemerality or sex biased dispersal or might also play a role. Experimental tests to investigate each of these possibilities need to be made directly.

A significant IBD relationship was found across: a) all regions analysed together, and b) across the eastern regions only, but these relationships were both nonlinear (Figure 4). The significant IBD result in the “all regions” analysis (Figure 4a) is probably a consequence of autocorrelation between the eastern regions and the spatially and genetically divergent north‐western region. Samples from the northern regions in eastern Australia were much more distinct genetically than anticipated from the short geographic distance that separates them from the populations further south (F_ST,_ Table 3). The lack of Asian lineage mtDNA haplotypes south of Bowen (Figure 1) is also evidence for introgression between the lineages having a greater impact on northern populations in eastern Australia than on those found further south. More admixture, and a wider distribution of mtDNA haplotype groups, would be expected across eastern Australia if mating was random and if both lineages had contributed equally to the local gene pool in northern Queensland.

### 4.2 Ecological differentiation in *Nezara viridula* and speciation in generalist insects

The extent to which the Asian and European lineages of *N. viridula* are ecologically differentiated with respect to one another is still unclear and requires experimental investigation. The genetics results we present provide the framework from which sound ecological tests can be designed and conducted in Australia. Some of the differences documented across *N. viridula* populations and across localities (Aldrich et al., 1987; Jeraj & Walter, 1998; Kon et al., 1988; Miklas et al., 2000; Panizzi & Meneguim, 1989; Ryan et al., 1995, 1996; Todd, 1989; Virant‐Doberlet et al., 2000) may well be associated with the two different lineages or their admixed populations. The differences in sexual sound communication across Australian and Slovenian populations of *N. viridula*, for example, could be explained partly by admixture between the Asian and European lineages in Australia (Jeraj & Walter, 1998; Ryan et al., 1996). Inferences are, however, difficult to make without the ecology of each lineage and their admixed populations having been investigated independently.

Ecological differences across Asian and European lineage bugs may restrict individuals with different ancestry to particular regions in Australia, perhaps limiting contact to certain periods annually, and thus contribute to the reduced gene flow between them in Australia. Adaptations to temperature and humidity are the most promising avenue for further investigation given the distribution of mtDNA haplotypes in Australia (Figure 1) and the results of the climatic modelling (Figure 5). Although we focus on high temperature and high humidity as important climatic factors here, low temperatures and dry conditions could be equally limiting for Asian lineage bugs. The climatic analysis of Australian *N. viridula* presented here should be treated as a preliminary, given that it relies on mtDNA to determine the genetic background of the bugs, and admixture across the lineages in Australia means that the mtDNA haplotype of an individual does not relate it definitively to one lineage or the other. We therefore recommend that these results be used only as a basis for future experimental tests of ecological differences across the Asian and European lineages of *N. viridula*.

The climatic analysis results are supported by comparisons between site temperatures (Table S8) and the available experimental evidence. The mean maximum temperatures in the hottest months for the north‐western region exceed experimentally determined climatic envelopes for primarily European lineage *N. viridula* from southern Queensland and central New South Wales (Chanthy et al., 2015; Velasco & Walter, 1993). Further, extreme climate events may have temperature or humidity parameters in excess of these values. Average minimum and maximum temperatures in the hottest months for the two sites sampled in the north‐western region were 24.9/39.0°C (KUN) and 24.3/37.4°C (DAR), respectively (Table S8). Developmental and longevity experiments for *N. viridula* show that bugs from southern QLD and central NSW perform poorly in high temperature and high humidity conditions typical of the hottest months in the north‐western region (Table S8). Mortality was over 80% for adult *N. viridula* from southern QLD after three weeks under alternating 27/37°C degree conditions and no adults survived to reproduce (Velasco & Walter, 1993). Mean longevity of *N. viridula* adults from central NSW dropped dramatically to 17.4 days at 36°C and 40% humidity and no mating was observed, with high humidity (80%) mean longevity was further reduced to 8.6 days (Chanthy et al., 2015). Experiments investigating interactions between life‐history parameters and climatic factors need to be conducted for primarily Asian lineage bugs from north‐western Australia and for admixed populations in northern Queensland.

Comparison across *N. viridula* and other *Nezara* species offers further support for climatic differences across lineages within this genus. *Nezara antennata*, the nearest relative of *N. viridula,* is adapted to cooler climates than *N. viridula* (Musolin, 2012; Tougou, Musolin, & Fujisaki, 2009; Yukawa et al., 2007), and given the similarities in host range and mating between these two species, adaptation to different climates may have been the common precursor to speciation in this group. Climatic tolerances might be one important component of speciation for host–plant generalists more broadly (Brunner & Frey, 2010; Gikonyo et al., 2017; Hereward et al., 2017). In Australia, *N. viridula* from eastern regions experience much cooler conditions and more variable photoperiods than those in the north‐western region (Table S8). Selection may have acted differently on the seasonal ecology of the Asian and European lineages of *N. viridula* in Australia and across populations in their original distributions. Diapause behaviour, for example, is unlikely to have been under the same selection pressures in tropical regions with climatic and photoperiodic similarity to the north‐western region in Australia. In Japan, *N. viridula* has undergone a range expansion into cooler regions (Musolin, Tougou, & Fujisaki, 2011; Tougou et al., 2009; Yukawa et al., 2007) and this pattern may, in part, be because European lineage bugs invaded that country.

With respect to the host plant associations of *N. viridula* in Australia, no host‐associated genetic structure was evident within any of the regions sampled (STRUCTURE, Figure 1, and PCA, Figure 3), so there is no evidence for host‐specific populations in Australia. Host plants vary region to region, however, and a true comparative analysis of host interactions among lineages is not possible with the data presented here. The process of speciation in generalist herbivorous insects, and the role that host plants play in this, has received less attention than in their specialist counterparts, and few studies investigate the population genetics of closely related generalist species while accounting for their host use (Vidal, Quinn, Stireman, Tinghitella, & Murphy, 2019). As a case study, *N. viridula* and its close relatives are likely to provide insight into speciation and host use evolution when insect–host relationships are truly general.

### 4.3 Implications for management

Two genetic populations of *N. viridula* are present in Australia (Figure 1) that should be treated separately ecologically and with respect to pest management and research. However, populations of *N. viridula* from eastern regions of Australia can be treated as a single interbreeding population, even though the biology of individuals in northern regions may be affected more strongly by introgression between the Asian and European lineages (Figure 1 and Table 3). Any ecological differences between Asian and European lineage *N. viridula* will influence the local pest ecology of this bug in Australia and internationally, but thorough experimental and genetic investigation of *N. viridula* is required to establish whether there are any consistent ecological differences between them.

The possibility of climatic differences between the lineages has serious consequences for the geographic range of *N. viridula* globally. It is unlikely to be coincidental that the two genetically different populations of *N. viridula* in Australia are found separately in regions of Australia with different climatic regimes and that these genetic populations relate primarily to only one of the Asian and European lineages of *N. viridula*. Genetic differences are also relevant to quarantine concerns in countries where only one lineage or another is currently present, because a secondary invasion may extend the geographic range of the pest. In Australia, host plant availability, generation number and timing, and climatic pressures will differ between some regions irrespective of any genetic influences associated with the Asian and European lineages. Additionally, *Trichopoda* parasitoids eggs were not found on any *N. viridula* adults in the north‐western region during sampling despite them being common on bugs in eastern Australia (pers. obs.). Whether these parasitoids have not dispersed to the north‐western region, are not climatically suited to the region or are ineffective at parasitizing Asian lineage bugs remains to be investigated.

### 4.4 General conclusions

Secondary contact and admixture between the lineages indicates that they do not represent cryptic species. However, the incomplete mixing of the two lineages in Australia, and the potential ecological differences across them suggest that they could be subspecies. Future research on *N. viridula* should treat the Asian and European lineages of *N. viridula* independently so that questions about the ecology of these lineages can be resolved fully and ecological tests can be structured according to the genetic background of each population (Walter, 2003). Several questions require resolution with respect to *N. viridula* specifically. Do Asian and European lineages of *N. viridula* differ ecologically from one another, especially with respect to their climatic tolerance as the distribution of their mtDNA haplotypes across climatically different regions in Australia suggest? Does the presence of multiple lineages of this bug make them more significant pests, or more widespread, than a single lineage? Has secondary contact between the Asian and European lineages of *N. viridula* in Australia and Japan yielded the same outcome, with only the limited spread of mtDNA haplotypes outside a narrow area of overlap? Is the restricted distribution of the Asian and European mtDNA haplotype groups in Australia a result of lineage‐specific climatic adaptations, or is mating nonrandom between them?

Detecting cryptic diversity and multiple introductions of generalist insects is challenging because the features of their ecology that would normally be seen to signal their presence (such as interacting with novel plant species in the case of host plant specialists) can be difficult to discern. This point is illustrated by the situation that we have uncovered in *N. viridula*, where two divergent and previously allopatric lineages mate when in sympatry but are not completely admixed within their invasive range. Over time *N. viridula* in Australia may even become a single fully admixed population with traits from both lineages, but this has not happened yet despite probably over a century of both being in Australia (Clarke, 1992). Clarification of the behavioural and ecological process responsible for the genetic structure of Australian *N. viridula* is a priority for our understanding of this pest, and resolving this point will also contribute to our understanding of how independent lineages of generalist herbivores should be in general treated. In the meantime, treating the lineages of *N. viridula* independently of one another with respect to quarantine risk, pest management and ecological research seems the most prudent approach to dealing with the uncertainty still surrounding our understanding of this species.

## CONFLICT OF INTEREST

There are no competing interests to declare.

## AUTHOR CONTRIBUTIONS

All authors designed the research. DRB conducted the sampling, designed the primers, performed the laboratory work and analysed the data. JPH performed the next‐generation sequencing. All authors contributed to the interpretation and writing the paper.

## DATA AND MATERIALS AVAILABILITY

GenBank accession numbers were assigned to all sequences generated during this study (MT178821 to MT179300, MT183174 to MT183340 and MT187283 to MT187459). All microsatellite genotypes and individual gene sequences are hosted at UQ eSpace (https://doi.org/10.14264/uql.2019.6).

## Supporting information

Supplementary Material
