# Supplementary material for: Multiple invasions of a generalist herbivore—Secondary contact between two divergent lineages of Nezara viridula Linnaeus in Australia
Source: Evol Appl. 2020 Jun 22;13(8):2113–29. doi: 10.1111/eva.12971 (PMC7463329; doi:10.1111/eva.12971)
Supplement: Supplementary file 1 — Supplementary Material [file EVA-13-2113-s001.docx]

**Table S1.** Australian *Nezara viridula* samples that were sequenced and genotyped in this study. They are ordered by their sampling locality and defined by collection date, host plant(s), genes sequenced, and numbers genotyped for microsatellite loci (m). The site code links the information in this table to raw data files. For COI and Ef1α, numbers in brackets represent those individuals for which only a single direction was sequenced. Entries with multiple host plants represent sites where insects were collected from multiple host plants growing immediately next to one another, and so plants could not be sampled independently of one another. A dash (-) indicates that no samples from that host in that location were used in an analysis. Totals are COI: 480, Tubα1: 177, Ef1α: 56 (111), and m: 571.

| **Site code** | **Locality** | **Long.** | **Lat.** | **Date (dd-mm-yy)** | **Host plant/s** | **COI** | **Tubα1** | **Ef1α** | **m** |  |
| --- | --- | --- | --- | --- | --- | --- | --- | --- | --- | --- |
| GH01 | Kununurra | -15.720 | 128.691 | 08/*ix*/2014 | *Vigna unguiculata* subsp. *sesquipedalis* (snake bean) | 16 | - | - | 16 |  |
| KU01 | Kununurra | -15.720 | 128.691 | 08/*ix*/2014 | *Momordica charantia* (bitter melon) | 22 | 4 | 5 | 24 |  |
| KU02 | Kununurra | -15.652 | 128.707 | 09/*ix*/2014 | *Salvia hispanica* (chia) | 23 | 6 | (6) | 24 |  |
| KU03 | Kununurra | -15.652 | 128.707 | 09/*ix*/2014 | *Carthamus tinctorius* (safflower) | 8 | 6 | 2 (4) | 8 |  |
| KU04 | Kununurra | -15.692 | 128.722 | 10/*ix*/2014 | *Solanum lycopersicum* (tomato) | 1 | 1 | (1) | 1 |  |
| KU05 | Kununurra | -15.692 | 128.722 | 10/*ix*/2014 | *Phaseolus vulgaris* (green bean) | 1 | 1 | 1 | 1 |  |
| KU06 | Kununurra | -15.692 | 128.721 | 10/*ix*/2014 | *Zea mays* (sweet corn) | 15 | 6 | 3 (3) | 15 |  |
| KU07 | Kununurra | -15.692 | 128.721 | 10/*ix*/2014 | *Ipomoea batatas* (sweet potato) | 2 | 2 | (2) | 2 |  |
| KU08 | Kununurra | -15.652 | 128.708 | 11/*ix*/2014 | *Gossypium hirsutum* (cotton) | 6 | 6 | 5 | 25 |  |
| KU09 | Kununurra | -15.652 | 128.708 | 11/*ix*/2014 | *Oryza sativa* (rice) | 1 | 1 | 1 | 1 |  |
| KU10 | Kununurra | -15.617 | 128.753 | 10/*ix*/2014 | *Zea mays* (sweet corn) | 1 | - | - | 1 |  |
| DW01 | Darwin | -12.443 | 130.930 | 15/*ix*/2014 | *Momordica charantia* (bitter melon) | 14 | 6 | 4 (1) | 16 |  |
| DW02 | Darwin | -12.443 | 130.930 | 15/*ix*/2014 | *Solanum lycopersicum* (tomato) | 1 | 1 | (1) | 1 |  |
| DW03 | Darwin | -12.443 | 130.930 | 15/*ix*/2014 | *Abelmoschus esculentus* (okra) | 2 | 2 | (2) | 2 |  |
| DW04* | Darwin | -12.555 | 131.257 | 12/*v*/2015 | *Passiflora edulis* (passionfruit) | 4 | 4 | 2 (2) | 4 |  |
| LR01* | Lockhart | -12.800 | 143.316 | 20/*vii*/2015 | *Cucurbita* sp. | 1 | - | - | 2 |  |
| TV01 | Townsville | -19.285 | 146.822 | 29/*ix*/2014 | *Solanum lycopersicum* (tomato) | 21 | 6 | 3 (2) | 24 |  |
| GU01 | Giru | -19.571 | 147.137 | 31/*ix*/2014 | *Solanum nigrum* | 1 | - | - | 8 |  |
| BO01 | Bowen | -20.078 | 148.151 | 31/*ix*/2014 | *Solanum nigrum* | 12 | 6 | 3 (3) | 15 |  |
| BO02 | Bowen | -20.106 | 148.135 | 31/*ix*/2014 | *Ricinus communis* (castor oil plant) | 6 | 6 | 3 (3) | 7 |  |
| BO03 | Bowen | -20.106 | 148.135 | 31/*ix*/2014 | *Cleome gynandra* (tickweed) | 5 | 5 | 1 (4) | 5 |  |
| BO04 | Bowen | -20.010 | 148.189 | 01/*x*/2014 | *Gossypium hirsutum* (cotton) | 19 | 6 | 2 (2) | 33 |  |
| BI01 | Biloela | -24.381 | 150.496 | 06/*ii*/2015 | *Gossypium hirsutum* (cotton) | 14 | 6 | 3 (3) | 16 |  |
| BI02 | Biloela | -24.379 | 150.494 | 06/*ii*/2015 | *Cajanus cajun* (pigeon pea) | 11 | 6 | 3 (1) | 16 |  |
| BI03 | Biloela | -24.372 | 150.511 | 06/*ii*/2015 | *Medicago sativa* (lucerne) | 2 | 2 | (2) | - |  |
|  |  |  |  |  |  |  | Continued | | |  |
| **Table S1** continued | |  |  |  |  |  |  |  |  |  |
| BI04 | Biloela | -24.432 | 150.539 | 23/*xi*/2015 | *Medicago sativa* (lucerne) | 22 | - | - | - |  |
| EM01 | Emerald | -23.553 | 148.233 | 15/*xi*/2014 | *Vigna radiata* (mung bean), *Arachis hypogaea* (peanuts) | 5 | 6 | 3 (3) | 18 | |
| EM02 | Emerald | -23.578 | 148.178 | 15/*xi*/2014 | *Medicago sativa* (lucerne) | 3 | 3 | (3) | 1 | |
| EM03 | Emerald | -23.518 | 148.203 | 05/*ii*/2015 | *Cajanus cajun* (pigeon pea) | 18 | 6 | (6) | 20 | |
| EM04 | Emerald | -23.525 | 148.216 | 04/*ii*/2015 | *Vigna radiata* (mung bean) | 20 | 5 | 3 (2) | 20 | |
| DA01 | Dalby | -27.283 | 151.275 | 14/*iv*/2015 | *Cajanus cajun* (pigeon pea) | 16 | 6 | 1 (5) | 16 | |
| DA02 | Dalby | -27.283 | 151.275 | 14/*iv*/2015 | *Gossypium hirsutum* (cotton) | 16 | 6 | (6) | 16 | |
| DA03 | Dalby | -27.687 | 151.307 | 02/*ix*/2015 | *Medicago polymorpha, Raphanus raphanistrum*,  *Vicia sativa* subsp. *sativa* | 19 | - | - | 0 | |
| DA04 | Dalby | -27.367 | 151.244 | 01/*ix*/2015 | *Rapistrum rugosum* (turnipweed), *Xanthium* sp. (Noogoora burr) | 11 | - | - | - | |
| GA01 | Gatton | -27.541 | 152.337 | 23/*viii*/2015 | *Amaranthus* sp., *Brassica* sp., *Malva parviflora*, *Medicago polymorpha, Medicago sativa* | 23 | - | - | - | |
| GA02 | Gatton | -27.248 | 151.282 | 01/*ix*/2015 | *Rapistrum rugosum* (turnipweed) | 2 | - | - | - | |
| GA03 | Gatton | -27.799 | 152.106 | 11/*viii*/2015 | *Rapistrum rugosum* (turnipweed) | 4 | - | - | - | |
| NA02 | Narrabri | -30.305 | 149.657 | 03/*xii*/2014 | *Helianthus annuus* (sunflower) | 1 | 1 | (1) | 1 | |
| NA01 | Narrabri | -30.186 | 149.473 | 04/*xii*/2014 | *Vigna radiata* (mung bean) | 22 | 5 | 1 (5) | 24 | |
| BB01 | Narrabri | -30.542 | 150.010 | 04/*xii*/2014 | *Medicago sativa* (lucerne) | 21 | 6 | 1 (5) | 24 | |
| NA04 | Narrabri | -30.186 | 149.473 | 19/*ii*/2015 | *Gossypium hirsutum* (cotton) | 23 | 6 | 1 (4) | 24 | |
| BR01 | Breeza | -31.158 | 150.396 | 23/*vi*/2014 | *Rapistrum rugosum* (turnipweed), *Xanthium* sp. (Noogoora burr) | 10 | 6 | 1 (5) | 10 | |
| BR02 | Breeza | -31.222 | 150.468 | 05/*xii*/2014 | *Xanthium* sp. (Noogoora burr) | 10 | 6 | (5) | 10 | |
| BR03 | Breeza | -31.158 | 150.396 | 23/*vi*/2014 | *Urtica* sp. (stinging nettle) | 2 | - | - | 2 | |
| GR01 | Griffith | -34.419 | 146.363 | 24/*ii*/2015 | *Cajanus cajun* (pigeon pea) | 5 | 6 | (6) | 23 | |
| GR02 | Griffith | -34.419 | 146.363 | 24/*ii*/2015 | *Glycine max* (soybean) | 5 | 6 | 1 (4) | 8 | |
| GR04 | Griffith | -34.441 | 146.037 | 25/*ii*/2015 | *Medicago sativa* (lucerne) | 6 | 6 | 1 (3) | 25 | |
| DP02 | Griffith | -34.596 | 145.977 | 25/*ii*/2015 | *Cajanus cajun* (pigeon pea) | - | - | - | 15 | |
| HA01 | Hay | -34.472 | 144.753 | 25/*ii*/2015 | *Gossypium hirsutum* (cotton) | 2 | 2 | 1 (1) | 2 | |
| HA02 | Hay | -34.472 | 144.753 | 25/*ii*/2015 | *Cajanus cajun* (pigeon pea) | 5 | 6 | 4 (2) | 21 | |

**Table S2.** Source of all COI sequences and their inclusion in the phylogenetic analysis (labelled Tree). Only Australian individuals sequenced in both directions were used. Comparisons were also made with Australian sequences and a 348bp fragment that had been used in previous phylogenetic studies of *N. viridula* (labelled Comp.) (Kavar et al. 2006; Li et al. 2010) to ensure that no Australian individuals matched the African lineage haplotype. The sequence labelled ‘Other’ in the location column was found in *N. viridula* from Brazil, California, Greece, Guadeloupe, Iran, Italy, Japan, Madeira, and Slovenia. Unpublished citations refer to sequences that were obtained only from GenBank accessions. Species designations are unchanged from those provided by the sequence authors.

| **Species** | **No. of Seq.** | **Host Plant/s** | **Location** | **Analysis** | **GenBank**  **Accession No.** | **Source** |
| --- | --- | --- | --- | --- | --- | --- |
| *N. viridula* | 176 | Various (see Table S1) | Australia (see Table 1) | Tree | - | This study |
| *N. viridula* | 2 | Unknown | India | Tree | KY694980, KY694982 | Singh and Kaur unpublished |
| *N. viridula* | 1 | Unknown | India, Meghalaya | Tree | KX351397 | Kuotsu *et al.* unpublished |
| *N. viridula* | 3 | Unknown | Pakistan | Tree | KY835350, KY836589, KY843444 | Ashfaq *et al.* unpublished |
| *N. viridula* | 2 | Unknown | India | Tree | KX467339, KX467340 | Rakshit *et al.* unpublished |
| *N. viridula* | 2 | Unknown | French Polynesia | Tree | KX054046 - KX054048 | Ramage *et al.* (2017) |
| *N. viridula* | 1 | Unknown | China | Tree | KC155924 | Zhang *et al.* unpublished |
| *N. viridula* | 1 | Unknown | India, Assam | Tree | KT879871 | Rakshit and Jalali unpublished |
| *N. viridula* | 3 | Unknown | Canada | Tree | KU601564 - KU601566 | Dhami *et al.* (2016) |
| *N. viridula* | 1 | Unknown | India | Tree | KU163629 | Kaur and Sharma unpublished |
| *N. viridula* | 1 | Unknown | Canada | Tree | KF303511 | Gariepy *et al.* (2014) |
| *N. viridula* | 3 | *Carya illinoinensis* (pecan) | Georgia, USA | Tree | KJ642018 - KJ642020 | Brown unpublished |
| *N. viridula* | 1 | Unknown | Florida, USA | Tree | KR044112 | Gwiazdowski *et al.* (2015) |
| *N. viridula* | 1 | Unknown | Texas, USA | Tree | KR037758 | Gwiazdowski *et al.* (2015) |
| *N. viridula* | 4 | *Gossypium hirsutum* (cotton) | Georgia, USA | Tree | JX548492 - JX548495 | Tillman *et al.* (2015) |
| *N. viridula* | 3 | Unknown | India, Karnataka | Tree | KR028339 - KR028341 | Reetha unpublished |
| *N. viridula* | 1 | Unknown | India | Tree | HQ236460 | Tembe unpublished |
| *N. viridula* | 1 | *Lablab purpureus* | India, Tamil Nadu | Tree | KJ559399 | Karthika and Krishnaveni unpublished |
| *N. viridula* | 1 | Unknown | India | Tree | KJ408787 | Kaur and Sharma unpublished |
|  |  |  |  |  |  | Continued |
| **Table S2** continued | | | | | | |
| *N. viridula* | 1 | Unknown | India | Tree | GQ306225 | Tembe unpublished |
| *N. viridula* | 1 | Unknown | India | Tree | KJ866507 | Rakshit unpublished |
| *N. viridula* | 1 | Unknown | South Korea | Tree | GQ292245 | Jung *et al.* (2011) |
| *N. antennata* | 2 | Unknown | South Korea | Tree | GQ292247, GQ292248 | Jung *et al.* (2011) |
| *N. antennata* | 1 | Unknown | South Korea | Tree | KC135971 | Jung unpublished |
| *N. antennata* | 2 | Unknown | China | Tree | FJ418861, FJ418862 | Li *et al.* (2010) |
| *N. viridula* | 4 | Unknown | China | Comp. | FJ418856, FJ418857, FJ418858, FJ418859 | Li *et al.* (2010) |
| *N. viridula* | 1 | Unknown | Iran | Comp. | FJ418860 | Li *et al.* (2010) |
| *N. viridula* | 1 | Unknown | Other | Comp. | AY839161 | Kavar *et al.* (2006), Li *et al.* (2010) |
| *N. viridula* | 1 | Unknown | Madeira | Comp. | AY839162 | Kavar *et al.* (2006) |
| *N. viridula* | 1 | Unknown | Brazil | Comp. | AY839163 | Kavar *et al.* (2006) |
| *N. viridula* | 1 | Unknown | Brazil | Comp. | AY839164 | Kavar *et al.* (2006) |
| *N. viridula* | 1 | Unknown | China, Japan | Comp. | AY839165 | Kavar *et al.* (2006), Li *et al.* (2010) |
| *N. viridula* | 1 | Unknown | Japan | Comp. | From paper | Kavar *et al.* (2006) |
| *N. viridula* | 1 | Unknown | Botswana | Comp. | AY839167 | Kavar *et al.* (2006) |
| *N. viridula* | 1 | Unknown | Japan | Comp. | - | Muraji *et al.* (2001) |

**Table S3.** Locus name, forward (F) and reverse (R) primer sequence, repeat motif, approximate fragment size (in base pairs) and dye used for the 12 microsatellite loci developed for genotyping samples of the Asian and European lineages of *Nezara viridula*.

| **Locus** | **Primer sequence (5’-3’)** | **Motif** | **Approximate product size (bp)** | **Dye** |
| --- | --- | --- | --- | --- |
| NEZA01 | F- TTCCTCTGCCTAGGTGTGCT | AAT | 161 | 6-FAM |
|  | R- TTCAGGTTCGCCAAGTCTTT |  |  |  |
| NEZA02 | F- GAAACCGGATCATCTCAAGC | AAT | 150 | VIC |
|  | R- ATTATTTACGGAGTTGGCCG |  |  |  |
| NEZA03 | F- GTGGATCGGCTTGACAATCT | AGC | 140 | NED |
|  | R- TTAGTCCACCTCGAACCCAG |  |  |  |
| NEZA04 | F- TACTCATCATGGCTGCGTGT | AAG | 188 | PET |
|  | R- ACCATCTAAGCATGGAAGCG |  |  |  |
| NEZA05 | F- CTTGTCGTAGCCAGGGAATC | AAAT | 107 | 6-FAM |
|  | R- TGGCCATGAAGAATAACAACA |  |  |  |
| NEZA06 | F- GTCGATCATTTCAGGCCAAC | AAG | 261 | VIC |
|  | R- TTAGCTTCCGTAAAGCGAGTC |  |  |  |
| NEZA07 | F- GGCTGTACAATGCTGGTCTTC | AAAT | 212 | NED |
|  | R- ATAATTCATAAGTGTAATGTGCTTCAG |  |  |  |
| NEZA08 | F- GGAACAAACCAAACAGGTGAA | AGG | 182 | PET |
|  | R- GGTATTCTGGAGCAAGTGGC |  |  |  |
| NEZA09 | F- CATAAGGAGGTCGCACGAAT | ACAT | 119 | 6-FAM |
|  | R- GCAAATCTCAACCTATTGATTTATGT |  |  |  |
| NEZA10 | F- AGGGAAATGTTGTGGACCTG | AAT | 127 | VIC |
|  | R- GACCGAGATCACGCTCAATC |  |  |  |
| NEZA11 | F- AAAGGACCTCTTGGGCATCT | AAG | 187 | NED |
|  | R- TTGACGTGGGAATTGTCAGA |  |  |  |
| NEZA12 | F- AAATCGATGCCAGAAACCTG | ATC | 243 | PET |
|  | R- TTGCGAATCTGGAACGAAAT |  |  |  |

**Table S4.** Primers, annealing temperatures and references for the PCR protocols of the COI, EF1α, and Tubα1 gene regions.

| **Gene region** | **Primers** | **Annealing temp. (°C)** | **Reference** |
| --- | --- | --- | --- |
| COI | F-GGTCAACAAATCATAAAGATATTGG | 51 (40 cycles) | (Folmer *et al.* 1994) |
|  | R- TAAACTTCAGGGTGACCAAAAAATCA |  |  |
| NV-EF1α | F- ACGCACTTCTTGCCTTCACT | 61 (8 cycles) 59 (32 cycles) | This study |
|  | R- TGACAACCATACCTGGTTTCAAT |  |  |
| NV-Tubα1 | F- TTCCATTTTGACCACTCACACT | 51 (8 cycles) 47 (32 cycles) | This study |
|  | R- CCAGTTGGACACCAATCAACA |  |  |

**Table S5.** Below the diagonal are pairwise F_ST_s for all populations of Australian *Nezara viridula* with sample size 19 or greater (Table 1) and using 10 microsatellite loci, with loci NEZA05 and NEZA10 having been excluded for having moderately high null allele estimates (~10%, Table 2). The results of pairwise exact G-tests for genotypic differentiation are shown above the diagonal with levels of significance: * = 0.01 - 0.05, ** = 0.01 – 0.001, *** = >0.001. Values that changed from significant to not significant, and *vice versa,* with the exclusion of NEZA05 and NEZA10 are highlighted in grey.

|  | **KUN** | **DAR** | **TOW** | **BOW** | **EMRa** | **EMRb** | **BIL** | **DAL** | **GAT** | **NARa** | **NARb** | **BBA** | **BRZ** | **GRIa** | **GRIb** | **HAY** |
| --- | --- | --- | --- | --- | --- | --- | --- | --- | --- | --- | --- | --- | --- | --- | --- | --- |
| **KUN** | - | *** | *** | *** | *** | *** | *** | *** | *** | *** | *** | *** | *** | *** | *** | *** |
| **DAR** | 0.02 | - | *** | *** | *** | *** | *** | *** | *** | *** | *** | *** | *** | *** | *** | *** |
| **TOW** | 0.11 | 0.13 | - | * | ns | ** | ** | *** | *** | *** | *** | *** | *** | *** | *** | *** |
| **BOW** | 0.15 | 0.16 | 0.02 | - | ns | ns | ns | *** | *** | *** | *** | *** | *** | *** | *** | *** |
| **EMRa** | 0.15 | 0.17 | 0.01 | 0.00 | - | ns | ns | ns | ** | ns | * | ns | *** | *** | *** | ** |
| **EMRb** | 0.14 | 0.17 | 0.02 | 0.00 | 0.00 | - | ns | *** | *** | *** | *** | *** | *** | *** | *** | *** |
| **BIL** | 0.16 | 0.18 | 0.02 | 0.00 | -0.01 | 0.00 | - | * | * | * | * | *** | *** | *** | *** | *** |
| **DAL** | 0.21 | 0.23 | 0.06 | 0.03 | 0.01 | 0.04 | 0.01 | - | ns | ns | ns | * | ** | ** | *** | ns |
| **GAT** | 0.21 | 0.24 | 0.07 | 0.03 | 0.02 | 0.03 | 0.02 | 0.00 | - | ns | * | *** | *** | *** | ** | ns |
| **NARa** | 0.21 | 0.23 | 0.05 | 0.02 | 0.01 | 0.02 | 0.01 | 0.00 | 0.00 | - | ns | ns | *** | ** | ** | * |
| **NARb** | 0.21 | 0.25 | 0.05 | 0.03 | 0.01 | 0.03 | 0.01 | 0.01 | 0.02 | 0.01 | - | ** | * | *** | *** | ns |
| **BBA** | 0.20 | 0.23 | 0.07 | 0.03 | 0.01 | 0.03 | 0.02 | 0.01 | 0.02 | 0.00 | 0.01 | - | ** | *** | *** | * |
| **BRZ** | 0.28 | 0.33 | 0.13 | 0.09 | 0.06 | 0.09 | 0.07 | 0.02 | 0.04 | 0.02 | 0.02 | 0.03 | - | ns | ns | ** |
| **GRIa** | 0.27 | 0.32 | 0.13 | 0.08 | 0.05 | 0.08 | 0.07 | 0.02 | 0.03 | 0.02 | 0.03 | 0.02 | -0.01 | - | ns | ns |
| **GRIb** | 0.27 | 0.31 | 0.12 | 0.07 | 0.05 | 0.07 | 0.06 | 0.02 | 0.02 | 0.01 | 0.04 | 0.03 | 0.01 | 0.00 | - | * |
| **HAY** | 0.21 | 0.24 | 0.06 | 0.03 | 0.02 | 0.04 | 0.02 | 0.00 | 0.01 | 0.01 | 0.00 | 0.00 | 0.02 | 0.01 | 0.01 | - |

**Table S6.** The regional number of private alleles and their total abundance is shown for the north-western, northern QLD and southern NSW regions. The regions between northern QLD and southern NSW were excluded to make a clearer comparison with the north-western region.

| **Region** | **Total no. alleles** | **No. private alleles** | **Abundance of private alleles** |
| --- | --- | --- | --- |
| North-western |  | 15 | 185 |
| Northern QLD |  | 1 | 1 |
| Southern NSW |  | 0 | 0 |

**Table S7.** Allelic richness of microsatellite loci for genotyped *Nezara viridula* populations and haplotype diversity associated with Asian and European mtDNA lineages for the Ef1α and Tubα1 genes. Individuals genotyped at microsatellite loci are grouped into those from northern Australia (141 individuals from Kununurra and Darwin) and those populations from eastern Australia (383 individuals from all other regions but excluding populations LHR and GRU (Figure S1)). The motif length of the locus is shown in brackets following the locus name. The proportion of the total number of alleles for each group is show in brackets following the number of alleles for the northern and eastern regions. The total number of alleles found in all populations is also given. The specific base pair length of all alleles is shown in the final column, with a superscript 1 indicating alleles found only in northern populations, and a superscript 2 indicating alleles found only in eastern populations. The proportion of nuDNA haplotypes found in individuals with mtDNA belonging to the Asian and European lineages for the Ef1α and Tubα1 genes is also shown (Figure 2). Of the 177 individuals sequences at the Tubα1 gene region, 100 had mtDNA corresponding to the European lineage, 72 to the Asian lineage, and 5 were not sequenced at the COI gene region. Of the 167 individuals sequences at the EF1α gene region, 93 had mtDNA corresponding to the European lineage, 69 to the Asian lineage, and 5 were not sequenced at the COI gene region.

| **Locus** | **Northern** | **Eastern** | **Total** | **Allele size (no. base pairs)** |
| --- | --- | --- | --- | --- |
| NEZA01 (tri-) | 5 (1.00) | 3 (0.60) | 5 | 179^1^, 182, 185, 188, 191^1^ |
| NEZA02 (tri-) | 5 (0.83) | 4 (0.67) | 6 | 155^1^, 158^1^, 164, 167, 170^2^, 173 |
| NEZA03 (tri-) | 2 (1.00) | 1 (0.50) | 2 | 164, 167^1^ |
| NEZA04 (tri-) | 8 (1.00) | 5 (0.63) | 8 | 196, 199, 202, 205^1^, 208,211, 214^1^, 220^1^ |
| NEZA05 (tetra-) | 4 (1.00) | 3 (0.75) | 4 | 120, 124, 128, 136^1^ |
| NEZA06 (tri-) | 4 (0.80) | 4 (0.80) | 5 | 312, 315, 318, 321, 324^1^, 330^2^ |
| NEZA07 (tetra-) | 3 (0.60) | 5 (1.00) | 5 | 218, 226, 230^2^, 234, 238^2^ |
| NEZA08 (tri-) | 3 (1.00) | 2 (0.67) | 3 | 204, 207, 210^1^ |
| NEZA09 (tetra-) | 8 (1.00) | 4 (0.50) | 8 | 106^1^, 110, 118, 122^1^, 126, 130^1^, 134^1^, 138 |
| NEZA10 (tri-) | 5 (1.00) | 5 (1.00) | 5 | 137, 140, 143, 146, 149, 152 |
| NEZA11 (tri-) | 4 (1.00) | 4 (1.00) | 4 | 206, 209, 212, 215 |
| NEZA12 (tri-) | 3 (1.00) | 3 (1.00) | 3 | 262, 265, 268 |
| Average across loci | 4.5 (0.93) | 3.8 (0.79) | 4.8 | - |
| **Gene** | **Asian mtDNA** | **European mtDNA** | **Both lineages** |  |
| Ef1α (gene) | 11 (0.92) | 7 (0.58) | 12 |  |
| Tubα1 (gene) | 5 (1.00) | 3 (0.60) | 5 |  |

**Table S8.** Summary climatic data are shown from weather stations nearest to each sample sites. Where multiple weather stations were present the first station with 10+ years of recent data was chosen. Mean minimum and maximum temperatures are shown for the coldest and hottest months. Data were obtained from the Australian Bureau of Meteorology on the 19^th^ of December 2019.

| **Locality** | **Region** | **Mean min. temp. for coldest month (°C)** | **Mean min. temp. for hottest month (°C)** | **Mean max. temp. for coldest month (°C)** | **Mean max. temp. for hottest month (°C)** |
| --- | --- | --- | --- | --- | --- |
| KUN | North-western | 14.3 | 24.9 | 30.6 | 39.0 |
| DAR | North-western | 14.5 | 24.3 | 32.3 | 37.4 |
| LHR | Far North QLD | 19.3 | 23.8 | 27.2 | 32.2 |
| TOW | Northern QLD | 13.7 | 24.3 | 25.2 | 31.5 |
| GRU | Northern QLD | 11.8 | 22.8 | 25.2 | 32.1 |
| BOW | Northern QLD | 13.4 | 23.8 | 24.5 | 31.5 |
| EMR | Central QLD | 9.1 | 22.2 | 23.4 | 34.6 |
| BIL | Central QLD | 5.2 | 19.8 | 21.9 | 33.2 |
| DAL | Southern QLD | 4.1 | 18.5 | 18.7 | 32.0 |
| GAT | Southern QLD | 6.2 | 19.1 | 20.8 | 31.6 |
| NAR & BBA | Central NSW | 3.7 | 19.3 | 18.0 | 33.8 |
| BRZ | Central NSW | 4.8 | 19.0 | 16.2 | 32.2 |
| GRI | Southern NSW | 3.7 | 17.2 | 13.9 | 31.9 |
| HAY | Southern NSW | 3.5 | 16.7 | 15.1 | 33.1 |


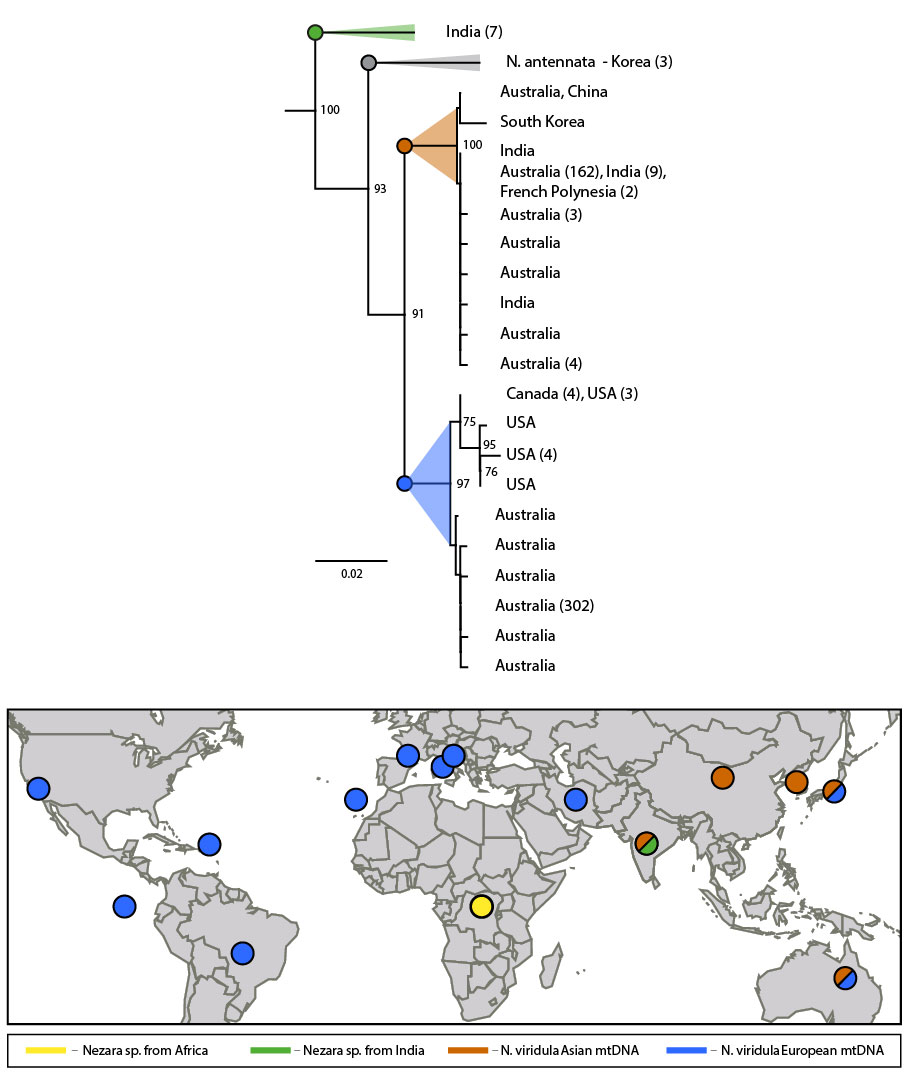
 **Figure S1.** Tree representing the phylogeny of *Nezara viridula*, based on a PhyML analysis using the a 557bp fragment of the COI gene region for *N. viridula* and *N. antennata* (Table S2)*.* Node labels are bootstrap support values and values below 70 are omitted. The map below shows the global distribution of the mtDNA haplotypes of *N. viridula* based on all known COI sequences (i.e. those included in Table S2). Only unique sequences were used and the number of samples that shared a sequence, and their origin, is shown in brackets (where it is greater than one). *Eurydema gebleri* (Pentatomidae) was included as the outgroup and was used to root the trees but is not shown. A comparison with sequences from the Kavar et al. (2006) data set shows that no Australia individuals had the African lineage haplotype.

**
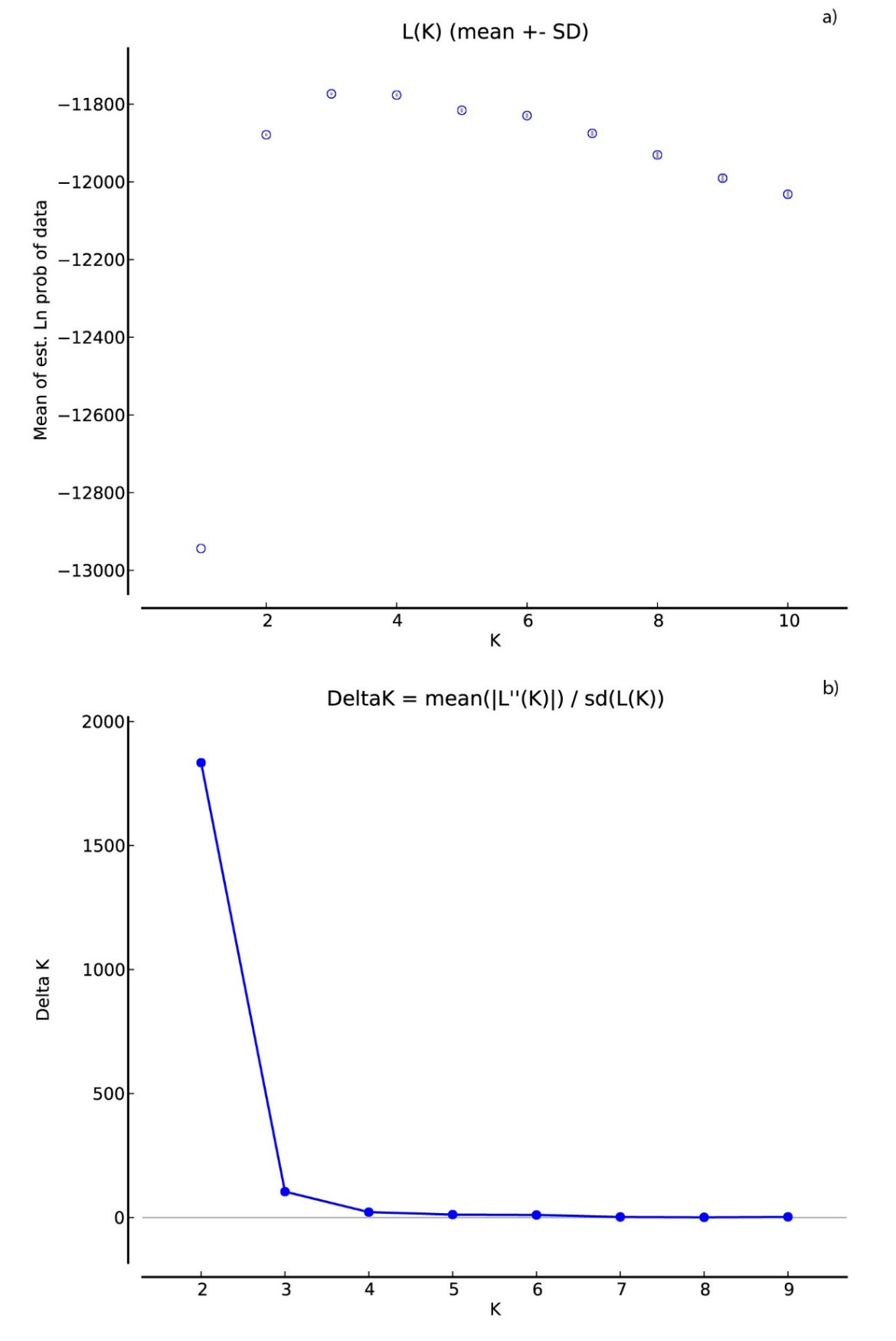
**

**Figure S2.** Output from STRUCTURE Harvester showing the likelihood of K (a) and Delta K (b). This indicates that K=2 is the most likely value of K.


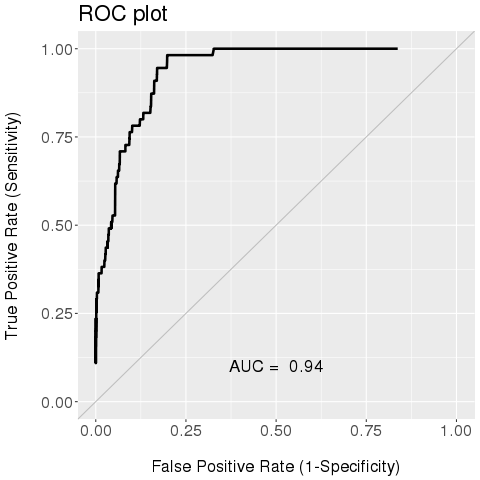


**Figure S3.** Sensitivity and specificity analysis for the European mtDNA group.


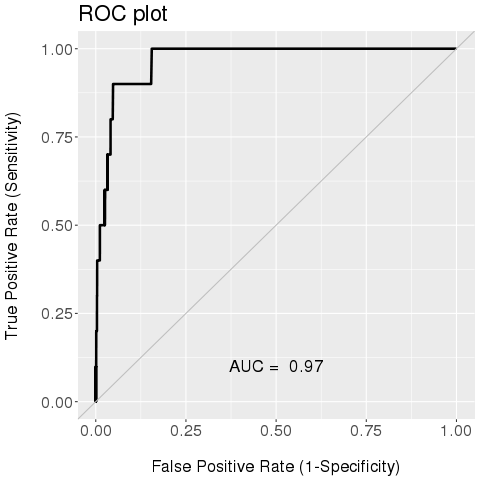


**Figure S4.** Sensitivity and specificity analysis for the Asian mtDNA group.


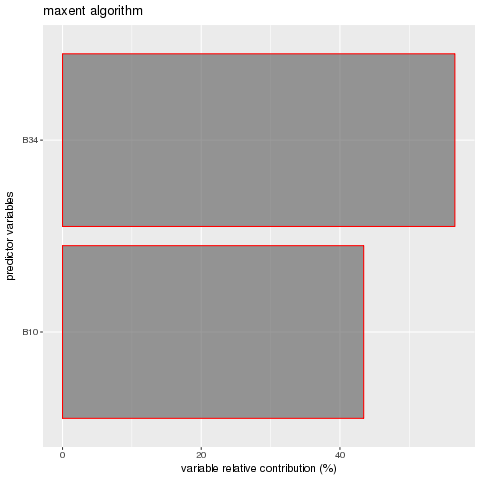


**Figure S5.** Relative contribution of the predictors for the European mtDNA group. B34 is mean moisture index for the warmest quarter and B10 is mean temperature for the warmest quarter.


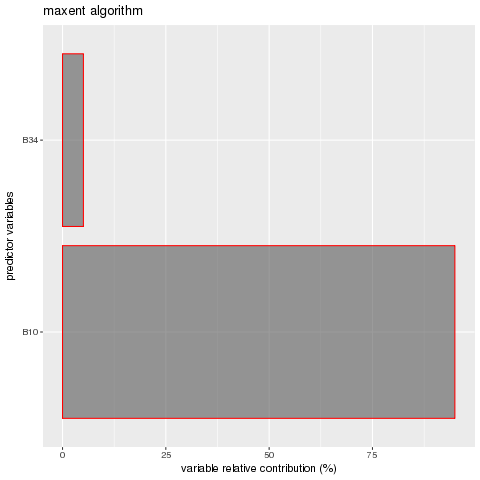


**Figure S6.** Relative contribution of the predictors for the Asian mtDNA group. B34 is mean moisture index for the warmest quarter and B10 is mean temperature for the warmest quarter.

**Supplementary Methods**

*DNA extraction protocols*

Salt extraction: The flight muscle of *Nezara viridula* was lysed using 3 µL of Proteinase K (20mg/ml) and 600 µL of lysis buffer (50mM tris-HCl pH 8.0, 20mM EDTA pH 8.0, and 2% SDS). The sample was incubated overnight at 55°C on a shaker, then at 37°C for 1 hour after 3 µL of Rnase (10mg/µL) had been added, and then placed on ice for 5 minutes. Each sample then had 200µL of NaCl (5M) added to it and was mixed through inversion before being centrifuged at high speed for 2 min. The supernatant was discarded and 1mL of cold isopropyl (100%) was added. The sample was repeatedly inverted (50x), centrifuged at high speed for 5 min, and washed twice using 1mL of EtOH (75%) (but left to rest for 15 min each time EtOH was added). The EtOH was removed a final time and the sample left to air dry overnight, when 50µL of TE buffer was added to dissolve the DNA pellet.

Chelex extraction: A single *N. viridula* leg was removed and placed in a 200µL of 20% Chelex (Bio-rad) solution. The solution was then heated to 98°C for 15 min.

*Microsatellite genotyping PCR protocol and primers*

PCR was carried out using 12 µL reactions with 1x myTAQ buffer, 0.1 µM of forward primer, 0.2 µM of reverse primer, 0.3 units of myTAQ HS DNA polymerase, 0.2 µM of M13 label (Schuelke 2000) (either 6-FAM, VIC, NED, or PET) and 3.0µl of DNA template. The primers used were those outlined above in Table S3. PCR cycling conditions were 10 min at 95°C followed by 25 cycles of 95°C for 25, 30 s at 57°C and 45 s at 72°C. A second series of 10 cycles followed, of 25 s at 95°C, 30 s at 54°C, 45 s at 72°C, and ending with an extension of 10 min at 72°C. The PCR product was cleaned using 2µl per sample of Exo-Ap (adapted from Werle et al. (1994)) with PCR conditions of 15min at 37°C followed by 15min at 80°C. Exo-Ap was made up of 5µl Exonuclease I (20U/µl), 10µl buffer and 85µl H2O combined with 20µl Antarctic Phosphatase (5U/µl), 10µl buffer and 70µl H2O. The products were separated by capillary electrophoresis on an ABI 3730XL (Macrogen, Korea).

*Gene sequencing PCR protocol and primers*

PCR reactions and protocols were the same for all three genes except that the annealing steps differed. PCR was carried out using 20 µL reactions with 1x myTAQ buffer, 0.4 µM of forward primer, 0.4 µM of reverse primer, 1 unit of myTAQ HS DNA polymerase, and 2.0µL of DNA template. The primers used were those outlined above in Table S4. PCR cycling conditions were 2 min at 95°C followed by 8 cycles of 95°C for 25, 1 min at 1st annealing temperature and 1 min 15 s at 72°C. A second series of 32 cycles followed, of 30 s at 95°C, 45 s at 2nd annealing temperature, 1 min 15 s at 72°C, and ending with an extension of 10 min at 72°C. The PCR product was cleaned using Exo-Ap (adapted from Werle et al. (1994)) as outlined above in the microsatellite PCR protocol. The products were then sequenced using Sanger sequencing (Macrogen, Korea).
